# Supplementary material for: Risk and protective factors for self-harm and suicide in children and adolescents: a systematic review and meta-analysis protocol
Source: BMJ Open. 2022 Nov 25;12(11):e058297. doi: 10.1136/bmjopen-2021-058297 (PMC9703327; doi:10.1136/bmjopen-2021-058297)
Supplement: Supplementary data [file bmjopen-2021-058297supp002.pdf]

Example of search strategy (MEDLINE):

1. "Suicid*".mp. (mp=AB,OX,HW,FX,OT,SY,KF,PX,TI,NM,RX,UI)
2. "self injurious behavio?r*".mp.
3. "non-suicid*".mp.
4. NSSI.mp.
5. "Parasuicid*".mp.
6. "Self-injur*".mp.
7. "self-cut*".mp.
8. "self-harm*".mp.
9. "Self-Mutilat*".mp.
10. "Autoaggress*".mp.
11. "Automutilat*".mp.
12. "Self-destruct*".mp.
13. "Self-immolat*".mp.
14. "Self-poison*".mp.
15. "Self-inflict*".mp.
16. "Kill ????self".mp.
17. "Kill ????selves".mp.
18. or/1-17
19. "p?ediatr*".mp.
20. "adolescen*".mp.
21. "teen*".mp.
22. "child*".mp.
23. "youth*".mp.
24. "young people".mp.
25. "kid*".mp.
26. "girl*".mp.
27. "boy*".mp.
28. "pupil*".mp.
29. or/19-28
30. "risk*".mp.
31. exp risk factor/
32. exp association/
33. "causal*".mp.
34. "correlat*".mp.
35. "relat*".mp.

36. "associat*".mp.
37. "predict*".mp.
38. "Advers*".mp.
39. "antecedent*".mp.
40. "Histor*".mp.
41. "?etiolog*".mp.
42. "protect*".mp.
43. "prevent*".mp.
44. "improv*".mp.
45. prevalence.mp.
46. incidence.mp.
47. "rate*".mp.
48. hazard.mp.
49. or/30-48
50. "Longitudinal*".mp.
51. Prospective.mp.
52. epidemiolog*.mp.
53. retrospective.mp.
54. trajector*.mp.
55. "case control*".mp.
56. "follow-up".mp.
57. "follow up".mp.
58. "follow* up".mp.
59. "cohort*".mp.
60. "wave*".mp.
61. "time*".mp.
62. "multi-wave*".mp.
63. or/50-61
64. exp suicide/
65. exp self injurious behavior/
66. exp Suicide, Attempted/
67. exp Suicide, Completed/
68. exp self mutilation/
69. or/64-68
70. exp child/

71. exp adolescent/
72. 70 or 71
73. exp epidemiologic methods/
74. limit 73 to yr=1971-1988
75. exp Case-Control Studies/
76. exp epidemiological studies/
77. exp Cohort Studies/
78. exp Controlled Before-After Studies/
79. exp Follow-Up Studies/
80. exp Longitudinal Studies/
81. exp "national longitudinal study of adolescent health"/
82. exp Prospective Studies/
83. exp Retrospective Studies/
84. exp observational study/
85. exp Historically Controlled Study/
86. exp Interrupted Time Series Analysis/
87. or/74-86
88. 18 or 69
89. 29 or 72
90. 63 or 87
91. 49 and 88 and 89 and 90
