# Supplementary material for: Risk and protective factors for self-harm and suicide in children and adolescents: a systematic review and meta-analysis protocol
Source: BMJ Open. 2022 Nov 25;12(11):e058297. doi: 10.1136/bmjopen-2021-058297 (PMC9703327; doi:10.1136/bmjopen-2021-058297)
Supplement: Author's manuscript [file bmjopen-2021-058297.draft_revisions.pdf]

BMJ Open

BMJ Open is committed to open peer review. As part of this commitment we make the peer review history of every article we publish publicly available.

When an article is published we post the peer reviewers' comments and the authors' responses online. We also post the versions of the paper that were used during peer review. These are the versions that the peer review comments apply to.

The versions of the paper that follow are the versions that were submitted during the peer review process. They are not the versions of record or the final published versions. They should not be cited or distributed as the published version of this manuscript.

BMJ Open is an open access journal and the full, final, typeset and author-corrected version of record of the manuscript is available on our site with no access controls, subscription charges or pay-per-view fees (http://bmjopen.bmj.com).

If you have any questions on BMJ Open's open peer review process please email info.bmjopen@bmj.com

BMJ Open

Risk and Protective Factors for Self-Harm and Suicide in Children and Adolescents: A Systematic Review and Meta-Analysis Protocol

Journal:	*BMJ Open*
Manuscript ID	bmjopen-2021-058297
Article Type:	Protocol
Date Submitted by the Author:	28-Feb-2022
Complete List of Authors:	Farbstein, Dan; Rambam Health Care Campus, Child and Adolescent Psychiatry; Technion Israel Institute of Technology The Ruth and Bruce Rappaport Faculty of Medicine,<br>Lukito, Steve; King's College London, Department of Child & Adolescent Psychiatry, Institute of Psychiatry, Psychology and Neuroscience<br>Yorke, Isabel; King's College London, Department of Child & Adolescent Psychiatry, Institute of Psychiatry, Psychology and Neuroscience<br>Bergou, Nicol; King's College London, Department of Psychosis Studies, Institute of Psychiatry, Psychology and Neuroscience<br>cliffe, charlotte; King's College London, Department of Biostatistics & Health Informatics, Institute of Psychiatry, Psychology and Neuroscience<br>Crudgington, Holly; King's College London, Department of Basic and Clinical Neurosciences; King's College London, Economic and Social Research Council (ESRC) Centre for Society and Mental Health<br>El-Aalem, Omar; South London & Maudsley NHS Foundation Trust<br>Itani, Lynn; Emirates Health Services<br>Owusu, Andy; South London & Maudsley NHS Foundation Trust<br>Sedgwick, Rosemary; South London and Maudsley NHS Foundation Trust<br>Singh, Nidhita; South London and Maudsley NHS Foundation Trust<br>Tarasenko, Anna; BrainPatch Ltd<br>Tucker, Gavin; South London & Maudsley NHS Foundation Trust<br>Wilson, Emma; King's College London, Department of Health Service and Population Research, Institute of Psychiatry, Psychology and Neuroscience; King's College London, Economic and Social Research Council (ESRC) Centre for Society and Mental Health<br>Woodhouse, Emma; Compass Psychology Services Ltd; King's College London<br>Epstein, Sophie; King's College London, Department of Psychological Medicine, Institute of Psychiatry, Psychology and Neuroscience<br>Ougrin, Dennis; King's College London, Department of Child & Adolescent Psychiatry, Institute of Psychiatry, Psychology and Neuroscience; Queen Mary University of London, Youth Resilience Research Unit, WHO Collaborating Centre for Mental Health Services Development
Keywords:	Suicide & self-harm < PSYCHIATRY, Child & adolescent psychiatry < PSYCHIATRY, Community child health < PAEDIATRICS

SCHOLARONE™
Manuscripts

Risk and Protective Factors for Self-Harm and Suicide in Children and Adolescents: A Systematic Review and Meta-Analysis Protocol

Dan Farbstein[1,2], Steve Lukito[3*], Isabel Yorke[3], Nicol Bergou[4], Charlotte Cliffe[5], Holly Crudgington[6,7], Omar El-Aalem[8], Lynn Itani[9], Andy Owusu[8], Rosemary Sedgwick[8], Nidhita Singh[8], Anna Tarasenko[10], Gavin Tucker[8], Emma Wilson[6,7], Emma Woodhouse[11, 12], Sophie Epstein[13‡], Dennis Ougrin[14,3 ‡]

[1]Child and Adolescent Psychiatry Unit, Psychiatric Division, Rambam Healthcare Campus, Haifa, Israel; [2]Ruth and Bruce Rappaport Faculty of Medicine, Technion – Israeli Institute of Technology, Haifa, Israel; [3]Department of Child & Adolescent Psychiatry, Institute of Psychiatry, Psychology and Neuroscience, King's College London, United Kingdom; [4]Department of Psychosis Studies, Institute of Psychiatry, Psychology and Neuroscience, King's College London, United Kingdom; [5]Department of Biostatistics & Health Informatics, Institute of Psychiatry, Psychology and Neuroscience, King's College London, United Kingdom; [6]Economic and Social Research Council (ESRC) Centre for Society and Mental Health, King's College London, United Kingdom; [7]Department of Health Service and Population Research, Institute of Psychiatry, Psychology and Neuroscience, King's College London, United Kingdom; [8]South London & Maudsley NHS Foundation Trust, United Kingdom; [9]Maudsley Health, Al Amal Psychiatric Hospital, Emirates Health Services, Dubai, United Arab Emirates; [10]BrainPatch Ltd., London, United Kingdom; [11]Compass Psychology Services Ltd., Bromley, United Kingdom; [12]Department of Forensic and Neurodevelopmental Sciences, Institute of Psychiatry, Psychology and Neuroscience, King's College London, United Kingdom; [13]Department of Psychological Medicine, Institute of Psychiatry, Psychology and Neuroscience, King's College London, United

For peer review only

Kingdom; [14]Youth Resilience Research Unit, WHO Collaborating Centre for Mental Health Services Development, Queen Mary University of London, United Kingdom.

‡ Senior authors contributed equally to this work.

*Correspondence: Steve Lukito, PhD.

King's College London

Institute of Psychiatry, Psychology and Neuroscience

Department of Child & Adolescent Psychiatry

De Crespigny Park

London SE5 8AF

Email: steve.lukito@kcl.ac.uk

For peer review only

Abstract:

Introduction: Self-harm and suicide are major public health concerns among children and adolescents. Many risk and protective factors for suicide and self-harm have been identified and reported in the literature. However, the capacity of these identified risk and protective factors to guide assessment and management is limited due to their great number. In this systematic review we aim to examine longitudinal studies of risk factors for self-harm and suicide in children and adolescents, to compare and rank the predictive ability of each risk factor and shed light on those that require further investigation.

Methods and Analysis: We performed a systematic search of EMBASE, PsychINFO, Medline, CINAHL and HMIC with no lower date limit. Additionally, we will contact experts in the field and search the reference lists of relevant reviews to retrieve any articles that were not identified in our search. We will extract relevant data and present a narrative synthesis, and combine the results into a meta-analyses if possible. We will assess the quality of studies using the Newcastle-Ottawa Scale, and present a summary of the quantity and the quality of the evidence for each risk or protective factor. Finally, we will attempt to rank the importance of the various risk factors for self-harm and suicide.

Ethics and dissemination: Ethical approval will not be sought as this is a systematic review of the literature. Results will be published in mental health journals and presented at conferences focused on suicide prevention.

PROSPERO registration number: CRD42021228212.

For peer review only

Strengths and limitations of this study

- This systematic review will examine data about risk and protective factors for suicide and self-harm in children and adolescents only from longitudinal studies, thus avoiding issues of temporality that exist in cross-sectional studies.
- We will perform a thorough and systematic search including several databases from health and mental health fields, searching citation lists of previous systematic reviews and contacting key authors in the field.
- Using the broad definition of self-harm (as opposed to differentiating between suicide attempt and non-suicidal self-injury) may create challenges in synthesising the data as its definition in each study may be different, but on the other hand will prevent us from missing important evidence on the subject.
- Examination of all risk and protective factors for self-harm and suicide in children and adolescents simultaneously in one systematic review will allow comparison of the importance of each factor.
- This systematic review will only include studies published in the English language and therefore may overlook risk and protective factors that are unique to individual countries or parts of the world.

For peer review only

Introduction:

Suicide and self-harm, defined as any act of self-poisoning or self-injury, regardless of the motivation[1] among adolescents and young people are of major public health concern. Acts of self-harm are known to be highly prevalent in adolescents. International community-based studies estimate that approximately ten to thirty percent of adolescents self-harm [2,3], and one to ten percent report to have made a suicide attempt at least once in their life [4–7]. These numbers are even higher in specific at-risk populations, such as those suffering from mental disorders, most notably depression and anxiety[6]. Most worryingly, self-harm and suicide attempts are some of the strongest predictive factors for completed suicide[8,9], which is now ranked as the second most common cause of death among 10-24 year olds, surpassed only by accidents[10].

Due to their great importance, many efforts have been undertaken to prevent self-harm and suicide attempts among adolescents. For example, a large international study examining school-based intervention programs for suicide prevention has showed that empowering youth and teaching coping skills results in decreased suicidal ideation and suicide attempts[11]. There have been recent advances in our understanding of precursors[12] and treatment[13] of self-harm in young people. However, the identification of adolescents at-risk for self-harm and suicide attempt remains difficult. Various studies and meta-analyses have been undertaken in an attempt to shed light on specific risk factors. Pre-existing externalizing and internalizing psychopathology have been demonstrated to be a significant risk factors for suicidal behavior[14,15]. Other risk factors include: sociodemographic factors, such as age[16], gender, sexual orientation, transgender status and socioeconomic status[17,18]; stressful life events, and most notably childhood abuse[19,20]; familial factors, including familial

For peer review only

psychopathology, family dysfunction, family conflict and perceived parental support [21–26]; school-related factors such as academic performance and school attendance[27,28]; biological factors such as genetic predisposition, changes in brain structure and function, and inflammatory status[29–31]; and social factors including peer-relatedness, bullying perpetration and victimization, and social media use[16,26,32–36]. Some studies have examined the relationship between suicidal behavior and specific emotions, such as hopefulness and self-esteem and emotional lability [16,35,37,38].

While these studies are of great importance in elucidating the risk factors for suicidal behavior, their clinical utility is limited. Such a large number of risk factors is likely to be overwhelming for the clinician meeting an adolescent in the emergency department, and thus not a helpful guide for the psychiatric examination. Additionally, many systematic reviews and meta-analyses take into account both cross-sectional studies and longitudinal studies. Cross-sectional studies may be highly informative about the associations between various exposures and outcomes. However, risk or protective factors that are examined in cross-sectional studies cannot predict future events, due to the unclear temporal precedence between predictors and outcomes.

This systematic review will therefore examine the evidence from longitudinal studies of all the known risk factors for self-harm and suicide in children and adolescents. This review will have two aims:

1. To provide a comparison of the predictive ability of the various risk factors for self-harm and suicide thus ranking their importance.
2. To examine risk factors for self-harm and suicide that are still debated in the literature, and thus to shed light on areas that are still in need of investigation.

For peer review only

Methods:

Our systematic review protocol follows the Preferred Reporting Items for Systematic Review and Meta-Analysis Protocols (PRISMA-P) guidelines (online supplementary file 1—PRISMA-P checklist) and is registered on PROSPERO, the international register of systematic reviews (ID CRD42021228212). Any changes to the protocol will be recorded on PROSPERO.

Eligibility Criteria:

1. *Study Population:* Our target population is children and adolescents, i.e., the population under the age of 18 at the time of both exposure to the risk factor and outcome (self-harm or suicide). The decision to choose 18 as the upper age limit was based on the fact that in most countries this is the age at which people transition to adulthood in many aspects. First, in many countries this is the age at which people complete school. Second, age 18 is when people legally become adults. Third, age 18 is the typical age at which the transition from child and adolescent to adult mental health services occurs.
2. *Types of studies to be included:* We will include only quantitative longitudinal studies, either prospective or retrospective. Thus, cross-sectional studies will be excluded. Retrospective studies that measure the existence and extent of risk factors using recall will be excluded, to avoid bias which tends to result in the exaggeration of the relationship between the risk factor and the outcome measured. Additionally, we will exclude intervention studies, qualitative studies, case reports or series, comments, editorials and conference abstracts.
3. *Exposure and outcome variables:* We will include any study that examines the relationship between any risk or protective factor and self-harm or suicide. Studies included must examine self-harm or suicide as an outcome. There are

For peer review only

many definitions and subdivisions for self-harm in the literature. As mentioned above, for our study we decided to use the UK/European definition of self-harm, which is: any act of self-poisoning or self-injury carried out by a person, irrespective of their motivation[39]. This broad definition includes both 1) non-suicidal self-injury: self-inflicted destruction of body tissues without any suicidal intent[40], and 2) suicide attempts: a nonfatal self-directed potentially injurious behavior with any intent to die as a result of the behavior[41]. Any type of ascertainment of the outcome variables is acceptable, including but not limited to self-report in questionnaires or interviews, collateral report, school or police records and medical files.

Search Methods:

References will be extracted from the following databases according to the search strategy that will be listed below:

- Medline – Ovid Platform
- PsycINFO – Ovid platform
- EMBASE – Ovid platform
- HMIC – Ovid platform
- CINAHL – EBSCO platform.

Databases will be searched with no lower limit date, up to the present.

For each of these databases, a search string was developed to include relevant keywords and synonyms (see "search strategy" in supplementary item 2). Examination of citation lists of previous relevant systematic reviews and meta-analyses will be conducted to retrieve any missed papers. Finally, experts in the field will be contacted to identify any missed papers.

Search strategy for electronic databases:

For peer review only

Search strategies were developed to include keywords and specific thesaurus terms for each database to include the following concepts:

1. Self-harm and suicide.
2. Populations under 18.
3. Association terms (e.g., relationship, risk factor, correlation, protective factors etc.)
4. Study designs.

To overcome linguistic variations, truncations and wildcards were employed when necessary. The list of keywords and thesaurus terms was based on previous systematic reviews about self-harm and suicide[27,36] and was discussed and finalized by a team of both experts in the field of self-harm research in children and adolescents and experts in the fields of systematic reviews and meta-analyses. The concept of "study designs" was introduced in other systematic reviews in this field[14], and thus was employed in our search as well. For this concept we used the keywords and thesaurus terms recommended by the *British Medical Journal*[42], but expanded the search to ensure sensitivity. Scoping searches were performed to refine the search strategy, and optimize the balance between sensitivity and specificity.

Reference Selection and Data Extraction:

Due to the large scope of our review, our research team includes 17 members. A document with screening instructions, detailing the inclusion and exclusion criteria for the title and abstract screen will be devised as follows: After producing a first draft of the screening instructions, the entire team will screen 100 references with each member blinded to the decision of the other team members. The decisions will be discussed together in team meetings, and the screening instruction will be revised accordingly. This process will be repeated until a 90% concordance rate is reached between all team

For peer review only

members. Following this process, references will be equally divided between all team members. Every reference will be screened by a single screener from the team, with a random sample of 10% of the references reviewed by a second screener. Agreement rates for every pair of screeners will be reported. Articles progressing to full-text screening will be retrieved and screened by a single screener, with a random sample of 10% reviewed by a second screener. All discarded items will be recorded, including a reason for exclusion at the full-text stage.

Data will be extracted by one reviewer, with a random sample of 10% of papers audited by a second reviewer. All data to be included in the meta-analysis will be extracted independently by two reviewers. Data will be recorded using a unified data extraction form that will be drafted a priori. The following data will be extracted: type of study, participant eligibility and recruitment method, participant description including age, gender and ethnicity, country, study dates, sample size, definitions and measures of outcomes, blinding, duration of follow-up, number and type of predictors, definition and method for measurement of candidate predictors, timing of predictor measurement, missing data and its handling, authors and year of publication. Any disagreement over reference eligibility or data extraction will be discussed between the two screeners/reviewers. If a consensus cannot be reached, a third screener (DO) will adjudicate.

Risk of bias (quality) assessment

Risk of bias assessment will be carried out by one reviewer, with a random sample of 10% assessed by a second reviewer. Assessment will be performed using the Newcastle-Ottawa Scale (NOS) for assessment of risk of bias in observational studies (cohort studies and case-control studies)[43]. In the case of discrepancies between the two reviewers regarding a study's quality, a third reviewer (DO) will adjudicate. Items

For peer review only

on the NOS scale will be adapted as necessary to allow better fit for the various risk and protective factors and outcomes that are measured.

Data presentation and statistical analysis:

First, included studies will be grouped according to the risk or protective factors that are described in them. We will attempt to group risk factors pertaining to a single subject and describe them collectively as well. For example: risk factor related to existing psychopathology, such as internalizing and externalizing psychopathology, and general existence of psychopathology; risk and protective factors relating to family relationships, such as family dynamics, parenting styles and familial resources; risk factors related to demographic variables, such as socioeconomic status, ethnicity, sexual orientation and gender identity; risk factors related to peer relationships, such as bullying and victimization, ostracization, and interactions via social media; previous self-harm events; and the predictive ability of suicide and self-harm scales. In each group, included studies will be described according to their characteristics in both text and tables. The results for each risk factor will be narratively synthesised. Where possible, studies will be grouped according to particular outcomes (for example: non-suicidal self-injury; suicide attempt; repeated self-harm) and exposures. Standardization of results will be performed where possible, and odds ratios will be calculated and reported.

Meta-analyses will be conducted if two or more studies examined similar exposures and outcomes and apply sufficiently similar methodologies. Since considerable heterogeneity is expected between studies, we will use a random effect model and present a summary of effect with 95% CI and P value. If possible, publication bias will be assessed by funnel plots and Egger's test.

For peer review only

For each exposure and outcome, an assessment of strength of evidence will be performed by applying the Grading of Recommendations Assessment, Development and Evaluation (GRADE) approach adapted for non-randomised studies. Subgroup analyses according to age, gender and ethnicity will be performed if possible. While all studies will be reported and included in the descriptive analysis regardless of the risk of bias as assessed by the NOS, this will be taken into account in terms of the strength of evidence within each group of studies: sensitivity analysis will be performed removing all studies which score below 60% on the NOS, and a synthesis of the evidence from the remaining studies will be reported to assess if this subtraction had an impact on the overall effect[44].

Patient and Public Involvement: Not applicable.

Discussion:

This systematic review and meta-analysis will attempt to outline the risk and protective factors for self-harm and suicide among children and adolescents. A major strength of our work will be its focus on longitudinal studies. The exclusion of cross-sectional studies will highlight the risk factors and protective factors that predict self-harm and suicide, as opposed to those that co-occur with them.

Conducting this systematic review and meta-analysis presents various challenges. Although we have used broad search terms to increase the chance of identifying all relevant data, there remains a chance that data could be missed due to the wide range of different terminology used in different settings. Additionally, it is possible that suicide or self-harm may not be reported in the title or abstract, and omitted from the keywords if it is not a major outcome in the study. To reduce the risk of missing significant data, in addition to database searches, we will examine the reference lists of previous systematic reviews and meta-analyses on the subject, and consult experts in

For peer review only

the field to identify additional papers. An additional challenge will be the synthesis of the data. Besides the variation in the quality of the studies that needs to be accounted for, methodology may differ significantly for the measurement of different risk and protective factors. For example, psychiatric symptoms may be measured using various methods: clinical evaluation, questionnaires and structured or semi-structured interviews, each method with its unique features in terms of reliability and validity. These variations would need to be taken into account when synthesising data of different studies.

The primary goal of our work will be to rank the significance of various risk and protective factors for self-harm and suicide in children and adolescents. We hope this work will inform the clinician assessing a child or adolescent in the outpatient clinic or the emergency department regarding important subjects that need to be covered in order to optimally assess future self-harm and suicide risk. The need for future research in different areas, whether due to insufficient studies, poor quality of studies, or the lack of use of similar terminology or methodology that would allow adequate data synthesis and conclusions, will also be highlighted.

Ethics and dissemination:

Ethical approval for this study is not needed, since it is based on the review of published literature only. The full protocol and the findings of this study will be published in peer-reviewed journals whose major audience are practitioners in child and adolescent mental health.

For peer review only

References:

1 Introduction and overview | Self-harm | Quality standards | NICE.

2 Brunner R, Kaess M, Parzer P, *et al.* Life-time prevalence and psychosocial correlates of adolescent direct self-injurious behavior: A comparative study of findings in 11 European countries. *J Child Psychol Psychiatry Allied Discip* 2014;**55**:337–48. doi:10.1111/jcpp.12166

3 Hawton K, Saunders KEA, O'Connor RC. Self-harm and suicide in adolescents. Lancet. 2012;**379**:2373–82. doi:10.1016/S0140-6736(12)60322-5

4 Nock MK, Green JG, Hwang I, *et al.* Prevalence, correlates, and treatment of lifetime suicidal behavior among adolescents: Results from the national comorbidity survey replication adolescent supplement. *JAMA Psychiatry* 2013;**70**:300–10. doi:10.1001/2013.jamapsychiatry.55

5 Madge N, Hewitt A, Hawton K, *et al.* Deliberate self-harm within an international community sample of young people: Comparative findings from the Child & Adolescent Self-harm in Europe (CASE) Study. *J Child Psychol Psychiatry Allied Discip* 2008;**49**:667–77. doi:10.1111/j.1469-7610.2008.01879.x

6 Moran P, Coffey C, Romaniuk H, *et al.* The natural history of self-harm from adolescence to young adulthood: A population-based cohort study. *Lancet* 2012;**379**:236–43. doi:10.1016/S0140-6736(11)61141-0

7 Kokkevi A, Rotsika V, Arapaki A, *et al.* Adolescents' self-reported suicide attempts, self-harm thoughts and their correlates across 17 European countries. J. Child Psychol. Psychiatry Allied Discip. 2012;**53**:381–9. doi:10.1111/j.1469-7610.2011.02457.x

8 Hawton K, Harriss L. Deliberate Self-Harm in Young People: Characteristics

For peer review only

and Subsequent Mortality in a 20-Year Cohort of Patients Presenting to Hospital. *J Clin Psychiatry* 2007;**68**:0–0.

9 Joiner TE, Conwell Y, Fitzpatrick KK, *et al.* Four studies on how past and current suicidality relate even when 'everything but the kitchen sink' is covaried. *J Abnorm Psychol* 2005;**114**:291–303. doi:10.1037/0021-843X.114.2.291

10 Patton GC, Coffey C, Sawyer SM, *et al.* Global patterns of mortality in young people: a systematic analysis of population health data. *Lancet* 2009;**374**:881–92. doi:10.1016/S0140-6736(09)60741-8

11 Wasserman D, Hoven CW, Wasserman C, *et al.* School-based suicide prevention programmes: The SEYLE cluster-randomised, controlled trial. *Lancet* 2015;**385**:1536–44. doi:10.1016/S0140-6736(14)61213-7

12 Baldwin JR, Arseneault L, Caspi A, *et al.* Adolescent Victimization and Self-Injurious Thoughts and Behaviors: A Genetically Sensitive Cohort Study. *J Am Acad Child Adolesc Psychiatry* 2019;**58**:506–13. doi:10.1016/J.JAAC.2018.07.903

13 Kothgassner OD, Robinson K, Goreis A, *et al.* Does treatment method matter? A meta-analysis of the past 20 years of research on therapeutic interventions for self-harm and suicidal ideation in adolescents. *Borderline Personal Disord Emot Dysregulation 2020 71* 2020;**7**:1–16. doi:10.1186/S40479-020-00123-9

14 Soto-Sanz V, Castellví P, Piqueras JA, *et al.* Internalizing and externalizing symptoms and suicidal behaviour in young people: a systematic review and meta-analysis of longitudinal studies. Acta Psychiatr. Scand. 2019;**140**:5–19. doi:10.1111/acps.13036

15 Melhem NM, Porta G, Oquendo MA, *et al.* Severity and variability of

For peer review only

depression symptoms predicting suicide attempt in high-risk individuals. *JAMA Psychiatry* 2019;**76**:603–12. doi:10.1001/jamapsychiatry.2018.4513

16 Borschmann R, Mundy LK, Canterford L, *et al.* Self-harm in primary school-aged children: Prospective cohort study. *PLoS One* 2020;**15**:e0242802. doi:10.1371/journal.pone.0242802

17 Miranda-Mendizabal A, Castellví P, Parés-Badell O, *et al.* Gender differences in suicidal behavior in adolescents and young adults: systematic review and meta-analysis of longitudinal studies. *Int J Public Health* 2019;**64**:265–83. doi:10.1007/s00038-018-1196-1

18 Di Giacomo E, Krausz M, Colmegna F, *et al.* Estimating the Risk of Attempted Suicide among Sexual Minority Youths. *JAMA Pediatr* 2018;**172**:1145–52. doi:10.1001/jamapediatrics.2018.2731

19 Gårdvik KS, Torgersen T, Rygg M, *et al.* Suicidality, function and associated negative life events in an adolescent psychiatric population at 3-year follow-up. *BMC Psychiatry* 2021;**21**. doi:10.1186/s12888-021-03100-w

20 Liu RT, Frazier EA, Cataldo AM, *et al.* Negative Life Events and Non-Suicidal Self-Injury in an Adolescent Inpatient Sample. *Arch Suicide Res* 2014;**18**:251–8. doi:10.1080/13811118.2013.824835

21 Brent DA, Melhem NM, Oquendo M, *et al.* Familial pathways to early-onset suicide attempt: A 5.6-year prospective study. *JAMA Psychiatry* 2015;**72**:160–8. doi:10.1001/jamapsychiatry.2014.2141

22 Adrian M, McCauley E, Berk MS, *et al.* Predictors and moderators of recurring self-harm in adolescents participating in a comparative treatment trial of psychological interventions. *J Child Psychol Psychiatry Allied Discip* 2019;**60**:1123–32. doi:10.1111/jcpp.13099

For peer review only

23 KING CA, SEGAL HG, NAYLOR M, *et al.* Family Functioning and Suicidal Behavior in Adolescent Inpatients with Mood Disorders. *J Am Acad Child Adolesc Psychiatry* 1993;**32**:1198–206. doi:10.1097/00004583-199311000-00013

24 Miller AB, Esposito-Smythers C, Leichtweis RN. Role of social support in adolescent suicidal ideation and suicide attempts. *J Adolesc Heal* 2015;**56**:286–92. doi:10.1016/j.jadohealth.2014.10.265

25 Kushal SA, Amin YM, Reza S, *et al.* Parent-adolescent relationships and their associations with adolescent suicidal behaviours: Secondary analysis of data from 52 countries using the Global School-based Health Survey. *EClinicalMedicine* 2021;**31**. doi:10.1016/j.eclinm.2020.100691

26 Victor SE, Hipwell AE, Stepp SD, *et al.* Parent and peer relationships as longitudinal predictors of adolescent non-suicidal self-injury onset. *Child Adolesc Psychiatry Ment Health* 2019;**13**:1. doi:10.1186/s13034-018-0261-0

27 Epstein S, Roberts E, Sedgwick R, *et al.* School absenteeism as a risk factor for self-harm and suicidal ideation in children and adolescents: a systematic review and meta-analysis. Eur. Child Adolesc. Psychiatry. 2019. doi:10.1007/s00787-019-01327-3

28 Orozco R, Benjet C, Borges G, *et al.* Association between attempted suicide and academic performance indicators among middle and high school students in Mexico: Results from a national survey. *Child Adolesc Psychiatry Ment Health* 2018;**12**:9. doi:10.1186/s13034-018-0215-6

29 Goldstein BI, Lotrich F, Axelson DA, *et al.* Inflammatory markers among adolescents and young adults with bipolar spectrum disorders. *J Clin Psychiatry* 2015;**76**:1556–63. doi:10.4088/JCP.14m09395

For peer review only

30 Johnston JAY, Wang F, Liu J, *et al.* Multimodal neuroimaging of frontolimbic structure and function associated with suicide attempts in adolescents and young adults with bipolar disorder. *Am J Psychiatry* 2017;**174**:667–75. doi:10.1176/appi.ajp.2016.15050652

31 Sarmiento-Hernández EI, Ulloa-Flores RE, Camarena-Medellín B, *et al.* Association between 5-HTTLPR polymorphism, suicide attempt and comorbidity in Mexican adolescents with major depressive disorder. *Actas Esp Psiquiatr* 2019;**47**:1–6.https://pubmed.ncbi.nlm.nih.gov/30724325/

32 Hawton K, Fagg J, Simkin S. Deliberate self-poisoning and self-injury in children and adolescents under 16 years of age in Oxford, 1976-1993. Br. J. Psychiatry. 1996;**169**:202–8. doi:10.1192/bjp.169.2.202

33 Zaborskis A, Ilionsky G, Tesler R, *et al.* The Association between Cyberbullying, School Bullying, and Suicidality among Adolescents: Findings from the Cross-National Study HBSC in Israel, Lithuania, and Luxembourg. Crisis. 2019;**40**:100–14. doi:10.1027/0227-5910/a000536

34 Van Geel M, Vedder P, Tanilon J. Relationship between peer victimization, cyberbullying, and suicide in children and adolescents ameta-analysis. *JAMA Pediatr* 2014;**168**:435–42. doi:10.1001/jamapediatrics.2013.4143

35 McMahon EM, Reulbach U, Corcoran P, *et al.* Factors associated with deliberate self-harm among Irish adolescents. *Psychol Med* 2010;**40**:1811–9. doi:10.1017/S0033291709992145

36 Sedgwick R, Epstein S, Dutta R, *et al.* Social media, internet use and suicide attempts in adolescents. Curr. Opin. Psychiatry. 2019;**32**:534–41. doi:10.1097/YCO.0000000000000547

37 Consoli A, Cohen D, Bodeau N, *et al.* Risk and Protective Factors for

For peer review only

Suicidality at 6-Month Follow-up in Adolescent Inpatients Who Attempted Suicide: An Exploratory Model. *Can J Psychiatry* 2015;**60**:S27–36.

38 Masi G, Milone A, Montesanto AR, *et al.* Non suicidal self-injury in referred adolescents with mood disorders and its association with cyclothymic-hypersensitive temperament. *J Affect Disord* 2018;**227**:477–82. doi:10.1016/j.jad.2017.11.049

39 Wilson E, Ougrin D. Commentary: Defining self-harm: how inconsistencies in language persist – a commentary/reflection on Ward and Curran (2021). *Child Adolesc Ment Health* Published Online First: 2021. doi:10.1111/CAMH.12502

40 About Self-Injury Archives - International Society for the Study of Self Injury. https://itriples.org/category/about-self-injury/ (accessed 13 May 2021).

41 Crosby AE, Ortega L, Melanson C. Self-directed violence surveillance: uniform definitions and recommended data elements. Atlanta, Georgia: 2011.

42 Study design search filters | BMJ Best Practice. https://bestpractice.bmj.com/info/toolkit/learn-ebm/study-design-search-filters/ (accessed 8 May 2021).

43 Wells G, Shea B, O'Connell D, *et al.* The Newcastle-Ottawa Scale (NOS) for assessing the quality of nonrandomised studies in meta-analyses. Ottawa Hosp. Res. Inst. http://www.ohri.ca/programs/clinical_epidemiology/oxford.asp (accessed 8 May 2021).

44 Epstein S, Roberts E, Sedgwick R, *et al.* Poor school attendance and exclusion: A systematic review protocol on educational risk factors for self-harm and suicidal behaviours. *BMJ Open* 2018;**8**:23953. doi:10.1136/bmjopen-2018-023953

For peer review only

Authors contribution:

Farbstein Dan: Drafting of manuscript, designing of study, search strategy, screening process and analysis.

Lukito Steve, Isabel Yorke: Designing of study, search strategy, screening process and analysis, critical revision and review of the protocol.

Nicol Bergou, Charlotte Cliffe, Holly Crudgington, Omar El-Aaleem, Lynn Itani, Andy Owusu, Rosemary Sedgwick, Nidhita Singh, Anna Tarasenko, Gavin Tucker, Emma Wilson, Emma Woodhouse: Designing of study, search strategy, screening process and analysis, revision and review of protocol.

Sophie Epstein, Dennis Ougrin: Designing of study, search strategy, screening process and analysis, critical revision and review of protocol.

Funding statement:

This work was supported by the joint UK Medical Research Foundation [MRF-058-0005-RG-OUGRI] and Medical Research Council (MRC) [MR/R004927/1] grant to DO, and the Daniel Turnberg Travel Fellowship [DTTFR12\1055] to DF. EW and HC were supported by the Economic and Social Research Council (ESRC) Centre for Society and Mental Health at King's College London [ES/S012567/1]. SE was funded by an MRC Clinical Research Training Fellowship [MR/T001437/1] and previously received salary support from an MQ Data Science Award and from the Psychiatry Research Trust. This work was also supported by funding from the UK Department of Health via the National Institute for Health Research (NIHR) Biomedical Research Centre (BRC) for Mental Health at South London (grant number N/A) and the Maudsley National Health Service (NHS) Foundation Trust and the IoPPN, King's College London (grant number N/A). The views expressed are those of the authors

and not necessarily those of the NHS, the NIHR, MRC, MRF, ESRC, or the Department of Health.

Competing interests:

The authors have no competing interests to disclose.

Word count: 2389

For peer review only

Reporting checklist for protocol of a systematic review and meta analysis.

Based on the PRISMA-P guidelines.

Instructions to authors

Complete this checklist by entering the page numbers from your manuscript where readers will find each of the items listed below.

Your article may not currently address all the items on the checklist. Please modify your text to include the missing information. If you are certain that an item does not apply, please write "n/a" and provide a short explanation.

Upload your completed checklist as an extra file when you submit to a journal.

In your methods section, say that you used the PRISMA-Preporting guidelines, and cite them as:

Moher D, Shamseer L, Clarke M, Ghersi D, Liberati A, Petticrew M, Shekelle P, Stewart LA. Preferred Reporting Items for Systematic Review and Meta-Analysis Protocols (PRISMA-P) 2015 statement. Syst Rev. 2015;4(1):1.

		Reporting Item	Page Number
**Title**			
Identification	#1a	Identify the report as a protocol of a systematic review	
Update	#1b	If the protocol is for an update of a previous systematic review, identify as such	
**Registration**			
	#2	If registered, provide the name of the registry (such as PROSPERO) and registration number	
**Authors**			
Contact	#3a	Provide name, institutional affiliation, e-mail address of all protocol authors; provide physical mailing address of corresponding author	

For peer review only

Contribution	#3b	Describe contributions of protocol authors and identify the guarantor of the review
**Amendments**		
	#4	If the protocol represents an amendment of a previously completed or published protocol, identify as such and list changes; otherwise, state plan for documenting important protocol amendments
**Support**		
Sources	#5a	Indicate sources of financial or other support for the review
Sponsor	#5b	Provide name for the review funder and / or sponsor
Role of sponsor or funder	#5c	Describe roles of funder(s), sponsor(s), and / or institution(s), if any, in developing the protocol
**Introduction**		
Rationale	#6	Describe the rationale for the review in the context of what is already known
Objectives	#7	Provide an explicit statement of the question(s) the review will address with reference to participants, interventions, comparators, and outcomes (PICO)
**Methods**		
Eligibility criteria	#8	Specify the study characteristics (such as PICO, study design, setting, time frame) and report characteristics (such as years considered, language, publication status) to be used as criteria for eligibility for the review
Information sources	#9	Describe all intended information sources (such as electronic databases, contact with study authors, trial registers or other grey literature sources) with planned dates of coverage
Search strategy	#10	Present draft of search strategy to be used for at least one electronic database, including planned limits, such that it could be repeated
Study records -	#11a	Describe the mechanism(s) that will be used to manage

For peer review only

data management		records and data throughout the review
Study records - selection process	#11b	State the process that will be used for selecting studies (such as two independent reviewers) through each phase of the review (that is, screening, eligibility and inclusion in meta-analysis)
Study records - data collection process	#11c	Describe planned method of extracting data from reports (such as piloting forms, done independently, in duplicate), any processes for obtaining and confirming data from investigators
Data items	#12	List and define all variables for which data will be sought (such as PICO items, funding sources), any pre-planned data assumptions and simplifications
Outcomes and prioritization	#13	List and define all outcomes for which data will be sought, including prioritization of main and additional outcomes, with rationale
Risk of bias in individual studies	#14	Describe anticipated methods for assessing risk of bias of individual studies, including whether this will be done at the outcome or study level, or both; state how this information will be used in data synthesis
Data synthesis	#15a	Describe criteria under which study data will be quantitatively synthesised
Data synthesis	#15b	If data are appropriate for quantitative synthesis, describe planned summary measures, methods of handling data and methods of combining data from studies, including any planned exploration of consistency (such as I2, Kendall's τ)
Data synthesis	#15c	Describe any proposed additional analyses (such as sensitivity or subgroup analyses, meta-regression)
Data synthesis	#15d	If quantitative synthesis is not appropriate, describe the type of summary planned
Meta-bias(es)	#16	Specify any planned assessment of meta-bias(es) (such as publication bias across studies, selective reporting within studies)

For peer review only

Confidence in cumulative evidence	#17	Describe how the strength of the body of evidence will be assessed (such as GRADE)

None The PRISMA-P elaboration and explanation paper is distributed under the terms of the Creative Commons Attribution License CC-BY. This checklist can be completed online using https://www.goodreports.org/, a tool made by the EQUATOR Network in collaboration with Penelope.ai

For peer review only

Example of search strategy (MEDLINE):

1. "Suicid*".mp. (mp=AB,OX,HW,FX,OT,SY,KF,PX,TI,NM,RX,UI)
2. "self injurious behavio?r*".mp.
3. "non-suicid*".mp.
4. NSSI.mp.
5. "Parasuicid*".mp.
6. "Self-injur*".mp.
7. "self-cut*".mp.
8. "self-harm*".mp.
9. "Self-Mutilat*".mp.
10. "Autoaggress*".mp.
11. "Automutilat*".mp.
12. "Self-destruct*".mp.
13. "Self-immolat*".mp.
14. "Self-poison*".mp.
15. "Self-inflict*".mp.
16. "Kill ????self".mp.
17. "Kill ????selves".mp.
18. or/1-17
19. "p?ediatr*".mp.
20. "adolescen*".mp.
21. "teen*".mp.
22. "child*".mp.
23. "youth*".mp.
24. "young people".mp.
25. "kid*".mp.
26. "girl*".mp.
27. "boy*".mp.
28. "pupil*".mp.
29. or/19-28
30. "risk*".mp.
31. exp risk factor/
32. exp association/
33. "causal*".mp.
34. "correlat*".mp.
35. "relat*".mp.

For peer review only

36. "associat*".mp.
37. "predict*".mp.
38. "Advers*".mp.
39. "antecedent*".mp.
40. "Histor*".mp.
41. "?etiolog*".mp.
42. "protect*".mp.
43. "prevent*".mp.
44. "improv*".mp.
45. prevalence.mp.
46. incidence.mp.
47. "rate*".mp.
48. hazard.mp.
49. or/30-48
50. "Longitudinal*".mp.
51. Prospective.mp.
52. epidemiolog*.mp.
53. retrospective.mp.
54. trajector*.mp.
55. "case control*".mp.
56. "follow-up".mp.
57. "follow up".mp.
58. "follow* up".mp.
59. "cohort*".mp.
60. "wave*".mp.
61. "time*".mp.
62. "multi-wave*".mp.
63. or/50-61
64. exp suicide/
65. exp self injurious behavior/
66. exp Suicide, Attempted/
67. exp Suicide, Completed/
68. exp self mutilation/
69. or/64-68
70. exp child/

For peer review only

71. exp adolescent/
72. 70 or 71
73. exp epidemiologic methods/
74. limit 73 to yr=1971-1988
75. exp Case-Control Studies/
76. exp epidemiological studies/
77. exp Cohort Studies/
78. exp Controlled Before-After Studies/
79. exp Follow-Up Studies/
80. exp Longitudinal Studies/
81. exp "national longitudinal study of adolescent health"/
82. exp Prospective Studies/
83. exp Retrospective Studies/
84. exp observational study/
85. exp Historically Controlled Study/
86. exp Interrupted Time Series Analysis/
87. or/74-86
88. 18 or 69
89. 29 or 72
90. 63 or 87
91. 49 and 88 and 89 and 90

For peer review only

BMJ Open

Risk and Protective Factors for Self-Harm and Suicide in Children and Adolescents: A Systematic Review and Meta-Analysis Protocol

Journal:	*BMJ Open*
Manuscript ID	bmjopen-2021-058297.R1
Article Type:	Protocol
Date Submitted by the Author:	23-Sep-2022
Complete List of Authors:	Farbstein, Dan; Rambam Health Care Campus, Child and Adolescent Psychiatry; Technion Israel Institute of Technology The Ruth and Bruce Rappaport Faculty of Medicine,<br>Lukito, Steve; King's College London, Department of Child & Adolescent Psychiatry, Institute of Psychiatry, Psychology and Neuroscience<br>Yorke, Isabel; King's College London, Department of Child & Adolescent Psychiatry, Institute of Psychiatry, Psychology and Neuroscience<br>Wilson, Emma; King's College London, Department of Health Service and Population Research, Institute of Psychiatry, Psychology and Neuroscience; King's College London, Economic and Social Research Council (ESRC) Centre for Society and Mental Health<br>Crudgington, Holly; King's College London, Department of Basic and Clinical Neurosciences; King's College London, Economic and Social Research Council (ESRC) Centre for Society and Mental Health<br>El-Aalem, Omar; South London and Maudsley NHS Foundation Trust<br>cliffe, charlotte; King's College London, Department of Biostatistics & Health Informatics, Institute of Psychiatry, Psychology and Neuroscience<br>Bergou, Nicol; King's College London, Department of Psychosis Studies, Institute of Psychiatry, Psychology and Neuroscience<br>Itani, Lynn; Maudsley Health, Al Amal Psychiatric Hospital, Emirates Health Services<br>Owusu, Andy; South London and Maudsley NHS Foundation Trust<br>Sedgwick, Rosemary; South London and Maudsley NHS Foundation Trust<br>Singh, Nidhita; South London and Maudsley NHS Foundation Trust<br>Tarasenko, Anna; BrainPatch Ltd<br>Tucker, Gavin; South London and Maudsley NHS Foundation Trust<br>Woodhouse, Emma; Compass Psychology Services Ltd; King's College London<br>Suzuki, Mimi; Queen Mary University of London & East London NHS Foundation Trust, Unit for Social and Community Psychiatry<br>Myerscough, Anna; Queen Mary University of London & East London NHS Foundation Trust, Unit for Social and Community Psychiatry<br>Lopez Chemas, Natalia; Queen Mary University of London & East London NHS Foundation Trust, Unit for Social and Community Psychiatry<br>Abdel-Halim, Nadia; Queen Mary University of London & East London NHS Foundation Trust, Unit for Social and Community Psychiatry<br>Del Giovane, Cinzia; University-Hospital of Modena and Reggio Emilia, Department of Medical and Surgical Sciences for Children and Adults; Institute of Primary Health Care (BIHAM), University of Bern

	Epstein, Sophie; King's College London, Department of Psychological Medicine, Institute of Psychiatry, Psychology and Neuroscience<br>Ougrin, Dennis; King's College London, Department of Child & Adolescent Psychiatry, Institute of Psychiatry, Psychology and Neuroscience; Queen Mary University of London, Youth Resilience Research Unit, WHO Collaborating Centre for Mental Health Services Development
**Primary Subject Heading**:	Paediatrics
Secondary Subject Heading:	Mental health
Keywords:	Suicide & self-harm < PSYCHIATRY, Child & adolescent psychiatry < PSYCHIATRY, Community child health < PAEDIATRICS

SCHOLARONE™ Manuscripts

Risk and Protective Factors for Self-Harm and Suicide in Children and Adolescents: A Systematic Review and Meta-Analysis Protocol

Dan Farbstein[1,2†], Steve Lukito[3*†], Isabel Yorke[3], Emma Wilson[4,5], Holly Crudgington[4,5], Omar El-Aalem[6], Nicol Bergou[7], Charlotte Cliffe[8], Lynn Itani[9], Andy Owusu[6], Rosemary Sedgwick[6], Nidhita Singh[6], Anna Tarasenko[10], Gavin Tucker[6], Emma Woodhouse[11, 12], Mimi Suzuki[13], Anna Louise Myerscough[13], Natalia Lopez Chemas[13], Nadia Abdel-Halim[13], Cinzia Del Giovane[14, 15], Sophie Epstein[16‡], Dennis Ougrin[17,3 ‡]

[1]Child and Adolescent Psychiatry Unit, Psychiatric Division, Rambam Healthcare Campus, Haifa, Israel;[2]Ruth and Bruce Rappaport Faculty of Medicine, Technion – Israeli Institute of Technology, Haifa, Israel;[3]Department of Child & Adolescent Psychiatry, Institute of Psychiatry, Psychology and Neuroscience, King's College London, United Kingdom;[4]Economic and Social Research Council (ESRC) Centre for Society and Mental Health, King's College London, United Kingdom; [5]Department of Health Service and Population Research, Institute of Psychiatry, Psychology and Neuroscience, King's College London, United Kingdom; [6]South London & Maudsley NHS Foundation Trust, United Kingdom; [7]Department of Psychosis Studies, Institute of Psychiatry, Psychology and Neuroscience, King's College London, United Kingdom; [8]Department of Biostatistics & Health Informatics, Institute of Psychiatry, Psychology and Neuroscience, King's College London, United Kingdom; [9]Maudsley Health, Al Amal Psychiatric Hospital, Emirates Health Services, Dubai, United Arab Emirates; [10]BrainPatch Ltd., London, United Kingdom; [11]Compass Psychology Services Ltd., Bromley, United Kingdom;[12]Department of Forensic and Neurodevelopmental Sciences, Institute of Psychiatry, Psychology and Neuroscience, King's College London, United Kingdom; [13] Unit for Social and Community Psychiatry, Queen Mary University of London & East London NHS Foundation Trust. [14]Department of Medical and Surgical Sciences for Children and Adults, University-Hospital of Modena and Reggio Emilia, Modena, Italy; [15]Institute of Primary Health Care (BIHAM), University of Bern, Switzerland, [16]Department of Psychological Medicine, Institute of Psychiatry, Psychology and Neuroscience, King's College London, United Kingdom; [17]Youth Resilience Research Unit, WHO Collaborating Centre for Mental Health Services Development, Queen Mary University of London, United Kingdom.

‡ Senior authors contributed equally to this work.

† Joint first author to this work

*Correspondence author: Steve Lukito, PhD.
King's College London

Institute of Psychiatry, Psychology and Neuroscience

Department of Child & Adolescent Psychiatry

De Crespigny Park

London SE5 8AF

Email: steve.lukito@kcl.ac.uk

For peer review only - http://bmjopen.bmj.com/site/about/guidelines.xhtml

Abstract:

Introduction: Self-harm and suicide are major public health concerns among children and adolescents. Many risk and protective factors for suicide and self-harm have been identified and reported in the literature. However, the capacity of these identified risk and protective factors to guide assessment and management is limited due to their great number. This protocol describes an ongoing systematic review and meta-analysis which aims to examine longitudinal studies of risk factors for self-harm and suicide in children and adolescents, to provide a comparison of the strengths of association of the various risk factors for self-harm and suicide and to shed light on those that require further investigation.

Methods and Analysis: We perform a systematic search of the literature using the databases EMBASE, PsycINFO, Medline, CINAHL and HMIC from inception up to the present. Additionally, we will contact experts in the field, including principal investigators whose peer-reviewed publications are included in our systematic review as well as investigators from our extensive research network, and we will search the reference lists of relevant reviews to retrieve any articles that were not identified in our search. We will extract relevant data and present a narrative synthesis and combine the results in meta-analyses where there are sufficient data. We will assess the risk of bias for each study using the Newcastle-Ottawa Scale and present a summary of the quantity and the quality of the evidence for each risk or protective factor.

Ethics and dissemination: Ethical approval will not be sought as this is a systematic review of the literature. Results will be published in mental health journals and presented at conferences focused on suicide prevention.

PROSPERO registration number: CRD42021228212.

For peer review only - http://bmjopen.bmj.com/site/about/guidelines.xhtml

Article Summary:

Strengths and limitations of this study

- This systematic review will examine data about risk and protective factors for suicide and self-harm in children and adolescents only from longitudinal studies, thus avoiding issues of temporality that exist in cross-sectional studies.
- We will perform a thorough and systematic search including several databases from health and mental health fields, searching reference lists of previous systematic reviews and contacting key authors in the field.
- Using the broad definition of self-harm (as opposed to differentiating between suicide attempt and non-suicidal self-injury) may create challenges in synthesising the data as its definition in each study may be different, but on the other hand will prevent us from missing important evidence on the subject.
- Examination of all identified risk and protective factors for self-harm and suicide in children and adolescents simultaneously in one systematic review will allow comparison of the importance of each factor.
- This systematic review will only include studies published in the English language and therefore may overlook risk and protective factors that are unique to individual countries or parts of the world.

For peer review only - http://bmjopen.bmj.com/site/about/guidelines.xhtml

For peer review only

Introduction:

Suicide and self-harm, defined as any act of self-poisoning or self-injury, regardless of the motivation [1] among adolescents and young people are of major public health concern. Acts of self-harm are known to be highly prevalent in adolescents. International community-based studies estimate that approximately ten to thirty percent of adolescents self-harm [2, 3], and one to ten percent report to have made a suicide attempt at least once in their life [4-7]. These numbers are even higher in specific at-risk populations, such as those with psychiatric disorders, most notably depression and anxiety [6]. Most worryingly, self-harm and suicide attempts are some of the strongest predictive factors for completed, or death by, suicide [8, 9], which is now ranked as the second most common cause of death among 10-24 year olds, surpassed only by accidents [10].

Due to their great importance, many efforts have been undertaken to prevent self-harm and suicide attempts among adolescents. For example, a large international study examining school-based intervention programs for suicide prevention has showed that empowering youth and teaching coping skills results in decreased suicidal ideation and suicide attempts [11]. There have been recent advances in our understanding of precursors [12] and treatment [13] of self-harm in young people. However, the identification of adolescents at-risk for self-harm and suicide attempt remains difficult. Various studies and meta-analyses have been undertaken in an attempt to shed light on specific risk factors for self-harm and suicide in this population. Pre-existing externalising (e.g., conduct problems) and internalising symptoms (e.g., depression or anxiety) of psychopathology have been demonstrated to be a significant risk factors for suicidal behaviour [14, 15]. Other risk factors include: sociodemographic factors, such as age [16], gender, sexual orientation, transgender status and socioeconomic status [17, 18]; stressful life events, and most notably childhood abuse [19, 20]; familial factors, including familial psychopathology, family dysfunction, family conflict and perceived parental support

For peer review only - http://bmjopen.bmj.com/site/about/guidelines.xhtml

[21-26]; school-related factors such as academic performance and school attendance [27, 28]; biological factors such as genetic predisposition, changes in brain structure and function, and inflammatory status [29-31]; and social factors including peer-relatedness, bullying perpetration and victimisation, and social media use [16, 26, 32-36]. Some studies have examined the relationship between self-harm or suicidal behaviour and specific emotions, such as hopefulness and self-esteem and emotional lability [16, 34, 37, 38].

While these studies are of great importance in elucidating the risk factors for self-harm and suicidal behaviour, their clinical utility is limited. Such a large number of risk factors is likely to be overwhelming for the clinician meeting an adolescent in the emergency department, and in the primary care setting, and thus not a helpful guide for the psychiatric examination. Additionally, many systematic reviews and meta-analyses take into account both cross-sectional studies and longitudinal studies [see, e.g., 18, 27, 36, 39, 40]. Cross-sectional studies may be highly informative about the associations between various exposures and outcomes. However, risk or protective factors that are examined in cross-sectional studies cannot predict future events, due to the unclear temporal precedence between predictors and outcomes.

This protocol describes an ongoing systematic review and meta-analysis which will examine the evidence from longitudinal studies of all identified risk factors for self-harm and suicide in children and adolescents. This review will have two aims:

1. To provide summary strengths of association of the various risk factors for self-harm and suicide.
2. To identify risk factors for self-harm and suicide that are still debated in the literature, and thus to shed light on areas that are still in need of investigation.

Methods:

For peer review only - http://bmjopen.bmj.com/site/about/guidelines.xhtml

Our systematic review protocol follows the Preferred Reporting Items for Systematic Review and Meta-Analysis Protocols (PRISMA-P) guidelines [41] (online supplementary file 1—PRISMA-P checklist). The reporting of the systematic review and meta-analysis resulting from this protocol will follow the PRISMA2020 guidelines [42], and a completed PRISMA2020 checklist will be submitted with the publication. The protocol is registered on PROSPERO, the international register of systematic reviews (ID CRD42021228212). Any changes to the protocol will be recorded on PROSPERO.

Eligibility Criteria:

1. *Study Population:* Our target population is male or female children (i.e., young persons aged up to 10 years) and adolescents (i.e., young persons aged 11-17 years in early, mid and late adolescence). The population must be under the age of 18 years at the time of both exposure to the risk factor and outcome (self-harm or suicide). The decision to choose 18 years as the upper age limit was based on the fact that in most countries this is the age at which people transition to adulthood in many aspects. First, in many countries this is the age at which people complete school. Second, age 18 is when people legally become adults. Third, age 18 is the typical age at which the transition from child and adolescent to adult mental health services occurs. If a study includes both young persons under 18 and young adults who are over 18, then the study will only be included if (1) the average age of participants is under 18 or (2) more than 50% of the participants are under 18 or (3) there is a subgroup analysis for participants in the study meeting the criteria (1) or (2).
2. *Types of studies to be included:* We will include only quantitative longitudinal studies, either prospective or retrospective. Thus, cross-sectional studies will be excluded. Retrospective studies that measure the existence and extent of risk factors using recall will be excluded, to avoid bias which tends to result in the exaggeration of the

For peer review only - http://bmjopen.bmj.com/site/about/guidelines.xhtml

relationship between the risk factor and the outcome measured. As we consider many different risk/protective factors altogether, we do not set a threshold of eligibility for follow-up time in this study. Additionally, we will exclude intervention studies, qualitative studies, case reports or series, commentaries, editorials and conference abstracts. Only papers written in English language will be included.

3. *Exposure and outcome variables:* We will include any study that examines the relationship between any risk or protective factor and self-harm or suicide. Studies included must examine self-harm or suicide as an outcome. There are many definitions and subdivisions for self-harm in the literature. As mentioned above, for our study we decided to use the UK/European definition of self-harm, which is: any act of self-poisoning or self-injury carried out by a person, irrespective of their motivation [43]. This broad definition includes both (1) non-suicidal self-injury: self-inflicted destruction of body tissues without any suicidal intent [44], and (2) suicide attempts: a nonfatal self-directed potentially injurious behaviour with any intent to die as a result of the behaviour [45]. Any type of ascertainment of the outcome variables is acceptable, including but not limited to self-report in questionnaires or interviews, collateral report, school or police records and medical files. As suicidal ideation is not our outcome of interest, studies that report on suicidal ideation as the only outcome will be excluded.

Search Methods:

References will be extracted from the following databases according to the search strategy described below:

- Medline – Ovid Platform
- PsycINFO – Ovid platform

For peer review only - http://bmjopen.bmj.com/site/about/guidelines.xhtml

- EMBASE – Ovid platform
- HMIC – Ovid platform
- CINAHL – EBSCO platform.

Databases will be searched with from inception, up to the present.

For each of these databases, a search string was developed to include relevant keywords and synonyms (see "search strategy" in supplementary item 2). Examination of citation lists of previous relevant systematic reviews and meta-analyses will be conducted to retrieve any missed papers. Finally, experts in the field, which will include principal investigators whose peer-reviewed publications are included in our systematic review, as well as investigators from our extensive immediate research network [e.g., 35, 46, 47, 48], will be contacted to identify any missed papers.

Search strategy for electronic databases:

Scoping searches were initially performed to refine the search strategy and to optimise the balance between sensitivity and specificity. Search strategies were subsequently developed to include keywords and specific thesaurus terms for each database to include the following concepts:

1. Self-harm and suicide.
2. Populations under 18.
3. Association terms (e.g., relationship, risk factor, correlation, protective factors etc.)
4. Study designs.

To overcome linguistic variations, truncations and wildcards were employed when necessary. The list of keywords and thesaurus terms was based on previous systematic reviews about self-harm and suicide [27, 35] and was discussed and finalised by a team of experts in the field of self-harm research in children and adolescents, tertiary- and secondary-level service clinicians,

For peer review only - http://bmjopen.bmj.com/site/about/guidelines.xhtml

and experts in the fields of systematic reviews and meta-analyses. The concept of "study designs" was introduced in other systematic reviews in this field [14], and thus was employed in our search as well. For this concept we used the keywords and thesaurus terms recommended by the *British Medical Journal Best Practice* to identify cohort and case-control studies [49], but expanded the search to ensure sensitivity.

Reference Selection and Data Extraction:

Our research team includes 22 team members, comprising a mixture of researchers, clinicians and clinical academics, including several with expertise in self-harm and several with extensive experience of systematic reviews and meta-analyses including a senior statistician. Following removal of duplicate references, a two-staged screening of references is undertaken aided by a pre-specified screening instruction document. The document, which details the inclusion and exclusion criteria is devised as follows: After producing a first draft of the screening instructions, the entire team will screen 100 references with each member blinded to the decision of the other team members. The decisions will be discussed together in team meetings, and any ambiguity or lack of clarity in the screening instruction will be addressed and specific clarifications added to the instructions as necessary. This process will be repeated until a 90% concordance rate is reached among all team members.

Due to the anticipated large number of references and the constraint of time and resources to conduct this large-scale systematic review, once ≥ 90% concordance has been reached during the initial calibration process, remaining references will be divided equally between all team members. Following this process, 10% of all references will be screened by two independent reviewers and references will be equally divided into sets and be distributed among all team members. Every set of references will be screened by one screener from the team during the title/abstract screening stage, with a random sample of 10% of the references reviewed by a

For peer review only

second screener, following similar approaches in previous large-scale systematic reviews [e.g., 50, 51, 52]. Agreement rates for every pair of screeners will be recorded and reported as percent agreement [53]. If the agreement rates between pairs of screeners were lower than 90% within the set of 10% randomly selected articles, all remaining articles in this set will be double-screened. Articles progressing to full-text screening will be retrieved and screened by a single screener, with a random sample of 10% reviewed by a second screener, again similar to previous approaches [50-52]. If we identify more than one study which includes the same or overlapping samples, we will prioritise studies using the following hierarchy: largest sample, longest follow-up, adjusted for highest number of confounders. All discarded items will be recorded, including a reason for exclusion at the full-text screening stage. These screening stages will be conducted using the online systematic review tool Rayyan (https://www.rayyan.ai/).

Data will be extracted using a data extraction form developed collaboratively and a priori. This will be piloted with the team and then for pragmatic reasons (due to the anticipated number of included studies) data on study characteristics will be extracted by one reviewer, with a random subsample of 10% of papers will be audited by a second reviewer. All statistical data relating to study results will be extracted by two independent reviewers. The following data will be extracted: type of study, participant eligibility and recruitment method, participant description including age, gender and ethnicity, diagnosis if applicable, country, study dates, sample size, definitions and measures of outcomes, blinding, duration of follow-up, number and type of predictors, definition and method for measurement of candidate predictors (e.g., categories may include demographics, psychopathology, family relationships, peer relationships, treatment history etc.), timing of predictor measurement, missing data and its handling, authors, year of publication, type of statistical analysis, summary statistics and summary findings.

For peer review only - http://bmjopen.bmj.com/site/about/guidelines.xhtml

With regard to the summary statistics, we will collect effect measures expressing the association between risk factors and outcomes, as reported in individual studies, which are likely to include odds ratios (*OR*), hazard ratios (*HR*), risk ratios (*RR*), mean difference (*MD*) and standardised mean difference (*SMD*) with 95% confidence intervals (*CI*) and significance (*p*) values, or standard errors where appropriate, as well as which covariates have been adjusted for.

Any disagreement over reference eligibility, during the title/abstract and full-text screening stages, or data extraction will be discussed between the two screeners/reviewers. If a consensus cannot be reached, a third screener (DO) will adjudicate.

Risk of bias assessment

The Newcastle-Ottawa Scale (NOS) [54] will be used to assess risk of bias in observational/non-randomised studies. The risk of bias assessment will be shared out amongst multiple members of the research team with pairs of reviewers working independently applying the NOS. Separate versions of NOS are available for assessing the risks of bias in case-control and cohort studies, based on (1) the selection of study participants, e.g., their representativeness and ascertainment; (2) the comparability between participant groups, i.e., cases/exposed cohort or controls/non-exposed cohort; and (3) the risk of bias associated with the exposures in cohort studies, i.e., the ascertainment of exposures, comparability of exposure ascertainment in case and controls, and response rates; or outcomes in cohort studies, i.e., the assessments of outcomes, follow-up periods, and adequacy of follow-up of cohorts. Assessment of risk of bias will be conducted separately for case-control and cohort studies using the appropriate NOS versions.

Certain items of the NOS require tailoring to the specific systematic review or meta-analysis being conducted. A version of the NOS scale adapted for relevance to this research question

For peer review only - http://bmjopen.bmj.com/site/about/guidelines.xhtml

has been developed and agreed a priori by the research team. In particular, we determined the acceptable level of loss to follow-up and acceptable methods of ascertainment of the exposure and outcome variables. Missing data within individual studies (e.g., due to attrition), will be reported and be taken into account as part of the quality assessment, and the level of bias it introduced into the findings will be considered for each study.

Finally, the discrepancies between two reviewers regarding a study's risk of bias ratings will be resolved through discussions, if necessary, in the presence of a third reviewer (DO) who will adjudicate if consensus cannot be reached.

Data presentation and statistical analysis:

Where possible a meta-analysis will be conducted to synthesise evidence. Alternatively, we will perform a narrative synthesis without meta-analysis using the quantitative data of the included studies [55]. Depending on the available data, synthesis of quantitative data may involve summarising estimates of effect (i.e., strength of association in our context) in narratives, or vote counting based on the direction of the effects [56]. We will calculate missing outcome data, if possible, from the available data within the article to enable consistent reporting or potentially to include them in the meta-analyses. If this is not possible, the findings will be reported within the narrative synthesis in qualitative manner. The findings of association between risk factors and self-harm outcomes will be grouped according to categories of outcomes (i.e., self-harm or completed suicide). Then, for each outcome group, the association between the risk factors and the outcome will be grouped by similar risk-factor concepts, in line with clinically meaningful categorisation from past literature, for instance 'existing psychopathology', including internalising, externalising, and general psychopathology; 'family relationships', including family dynamics, parenting styles and familial resources; 'demographic variables', including socioeconomic status, ethnicity, sexual

For peer review only - http://bmjopen.bmj.com/site/about/guidelines.xhtml

orientation and gender identity; 'peer relationships', such as bullying and victimisation, ostracisation, and interactions via social media; and previous self-harm events. The relative strength of associations between all identified factors with the outcome will be discussed. Extracted data about participant sample characteristics, study methodologies, and a summary of findings from each study will be presented in a table of characteristics of included studies.

Random-effect meta-analysis will be conducted, given the anticipated variation of sample characteristics and outcome measures across studies. There is likely to be variation in the statistical effect measures used across the literature, however, most studies are expected to compare those with and without the exposure of interest when estimating their association with binary self-harm outcomes (i.e., with or without self-harm), and would use *OR*s, *HR*s or *RR*s to index such association. *OR*s are expected to be the effect measure mostly used by any eligible case-control studies. Furthermore, the *OR*s can be easily interpreted clinically [57, 58]. For these reasons, synthesis will be conducted using *OR* summary statistics to examine the pooled association between each predictor and self-harm. Where possible, other effect measures will be transformed to *OR*s for synthesis.

The meta-analyses will be conducted on findings from studies investigating similar exposure and outcome variables. If a study reports multiple effects of interest, then we will include all the available effects. If there is more than one reported effect in relation to the same exposure and outcome, the effect adjusted for the largest number of confounders will be included. We will illustrate the data using a forest plot, with 95% *CI* and *p*-value reported. It is expected that there will be significant heterogeneity between studies. Thus, the I^2 statistics will be calculated to index statistical heterogeneity in the meta-analysis. Finally, publication bias will be assessed by funnel plots and Egger's test if there are at least ten studies within the meta-analysis (Cochrane Handbook 13.3.5.4; [59]).

For peer review only - http://bmjopen.bmj.com/site/about/guidelines.xhtml

For each exposure and outcome, an assessment of strength of evidence will be performed by applying the Grading of Recommendations Assessment, Development and Evaluation (GRADE; [60]) approach adapted for non-randomised studies. We will use the GRADE tool to assess the quality of evidence, i.e., the certainty of the estimate of association between individual risk factors and each self-harm outcome. The GRADE tool will only be applied following a meta-analysis, i.e., when the pooled evidence of association between specific risk factors and a self-harm outcome could be obtained. We will use the 95% *CI* to assess precision for the GRADE tool. If the 95% *CI* does not cross the line of non-significance, the risk estimate will be considered to be sufficiently precise.

Subgroup analyses according to age (children, i.e., young persons aged up to 10 years *vs.* adolescents, i.e., young persons aged 11-17 years), gender (female *vs.* male) and country (high- *vs.* low- and middle-income country) will be conducted if possible. The quantitative synthesis of findings will be based on meta-analyses of all samples collected in the study. However, to assess the influence of risk of bias of the individual studies in the quantitative synthesis, we also plan to conduct sensitivity meta-analyses including only studies deemed to have low risk of bias (see e.g. [61] for a similar approach). Studies with low risk of bias are defined by a total NOS score ≥ 7 following past reports [e.g., 62, 63].

Patient and Public Involvement: Not applicable.

Discussion:

This systematic review and meta-analysis will attempt to outline the risk and protective factors for self-harm and suicide among children and adolescents. A major strength of our work will be its focus on longitudinal studies. The exclusion of cross-sectional studies will highlight the risk factors and protective factors that predict self-harm and suicide, as opposed to those that co-occur with them.

For peer review only - http://bmjopen.bmj.com/site/about/guidelines.xhtml

Conducting this systematic review and meta-analysis presents various challenges. Although we have used broad search terms to increase the chance of identifying all relevant data, there remains a chance that data could be missed due to the wide range of different terminology used in different settings. Additionally, it is possible that suicide or self-harm may not be reported in the title or abstract and are omitted from the keywords if it is not a major outcome in the study. To reduce the risk of missing significant data, in addition to database searches, we will examine the reference lists of previous systematic reviews and meta-analyses on the subject and consult experts in the field (i.e., principal investigators of peer-reviewed papers included in this review as well as investigators from our research network) to identify additional papers. An additional challenge will be the synthesis of the data. Besides the variation in the level of bias in the studies that needs to be accounted for, methodology may differ significantly for the measurement of different risk and protective factors. For example, psychiatric symptoms may be measured using various methods: clinical evaluation, questionnaires and structured or semi-structured interviews, each method with its unique features in terms of reliability and validity. These variations will need to be taken into account when synthesising data of different studies.

The primary goal of our work will be to describe the strength of association between various risk and protective factors and self-harm and suicide outcomes in children and adolescents. We hope this work will inform the clinician assessing a child or adolescent in the outpatient clinic or emergency department regarding important areas that need to be covered to optimally assess future self-harm and suicide risk. The need for future research in different areas, whether due to insufficient studies, poorly conducted studies, or the lack of use of similar terminology or methodology that would allow adequate data synthesis and conclusions, will also be highlighted.

Ethics and dissemination:

For peer review only - http://bmjopen.bmj.com/site/about/guidelines.xhtml

1 Ethical approval for this study is not needed, since it is based on the review of published literature only. The full protocol and the findings of this study will be published in peer-reviewed journals whose major audience are practitioners in child and adolescent mental health.

For peer review only

For peer review only - http://bmjopen.bmj.com/site/about/guidelines.xhtml

Authors contributions:

Dan Farbstein, Steve Lukito: Drafting of manuscript, designing of study, search strategy, screening process and analysis.

Isabel Yorke: Designing of study, search strategy, screening process and analysis, critical revision and review of the protocol.

Emma Wilson, Holly Crudgington, Omar El-Aalem, Nicol Bergou, Charlotte Cliffe, Lynn Itani, Andy Owusu, Rosemary Sedgwick, Nidhita Singh, Anna Tarasenko, Gavin Tucker, Emma Woodhouse: Designing of study, search strategy, screening process and analysis, revision and review of protocol.

Mimi Suzuki, Anna Louise Myerscough, Natalia Lopez Chemas, Nadia Abdel-Halim: Screening process and analysis, revision and review of protocol.

Cinzia Del Giovane, Sophie Epstein, Dennis Ougrin: Designing of study, search strategy, screening process and analysis, critical revision and review of protocol.

Funding statement:

This work was supported by the joint UK Medical Research Foundation [MRF-058-0005-RG-OUGRI] and Medical Research Council (MRC) [MR/R004927/1] grant to DO, and the Daniel Turnberg Travel Fellowship [DTTFR12\1055] to DF. EW and HC were supported by the Economic and Social Research Council (ESRC) Centre for Society and Mental Health at King's College London [ES/S012567/1]. SE was funded by an MRC Clinical Research Training Fellowship [MR/T001437/1] and previously received salary support from an MQ Data Science Award and from the Psychiatry Research Trust. This work was also supported by funding from the UK Department of Health via the National Institute for Health Research

For peer review only - http://bmjopen.bmj.com/site/about/guidelines.xhtml

(NIHR) Biomedical Research Centre (BRC) for Mental Health at South London (grant number N/A) and the Maudsley National Health Service (NHS) Foundation Trust and the IoPPN, King's College London (grant number N/A). The views expressed are those of the authors and not necessarily those of the NHS, the NIHR, MRC, MRF, ESRC, or the Department of Health.

For peer review only

Competing interests:

The authors have no competing interests to disclose.

Word count: 3554

References:

1. National Institute for Health and Care Excellence (NICE), *Self-harm [NICE Quality Standard No. 34].* 2019.
2. Brunner, R., et al., *Life-time prevalence and psychosocial correlates of adolescent direct self-injurious behavior: a comparative study of findings in 11 European countries.* J Child Psychol Psychiatry, 2014. **55**(4): p. 337-48.
3. Hawton, K., K.E. Saunders, and R.C. O'Connor, *Self-harm and suicide in adolescents.* Lancet, 2012. **379**(9834): p. 2373-82.
4. Madge, N., et al., *Deliberate self-harm within an international community sample of young people: comparative findings from the Child & Adolescent Self-harm in Europe (CASE) Study.* J Child Psychol Psychiatry, 2008. **49**(6): p. 667-77.

For peer review only - http://bmjopen.bmj.com/site/about/guidelines.xhtml

5. Nock, M.K., et al., *Prevalence, correlates, and treatment of lifetime suicidal behavior among adolescents: results from the National Comorbidity Survey Replication Adolescent Supplement.* JAMA Psychiatry, 2013. **70**(3): p. 300-10.

6. Moran, P., et al., *The natural history of self-harm from adolescence to young adulthood: a population-based cohort study.* Lancet, 2012. **379**(9812): p. 236-43.

7. Kokkevi, A., et al., *Adolescents' self-reported suicide attempts, self-harm thoughts and their correlates across 17 European countries.* J Child Psychol Psychiatry, 2012. **53**(4): p. 381-9.

8. Hawton, K. and L. Harriss, *Deliberate self-harm in young people: characteristics and subsequent mortality in a 20-year cohort of patients presenting to hospital.* J Clin Psychiatry, 2007. **68**(10): p. 1574-83.

9. Joiner, T.E., Jr., et al., *Four studies on how past and current suicidality relate even when "everything but the kitchen sink" is covaried.* J Abnorm Psychol, 2005. **114**(2): p. 291-303.

10. Patton, G.C., et al., *Global patterns of mortality in young people: a systematic analysis of population health data.* Lancet, 2009. **374**(9693): p. 881-92.

11. Wasserman, D., et al., *School-based suicide prevention programmes: the SEYLE cluster-randomised, controlled trial.* Lancet, 2015. **385**(9977): p. 1536-44.

12. Baldwin, J.R., et al., *Adolescent Victimization and Self-Injurious Thoughts and Behaviors: A Genetically Sensitive Cohort Study.* J Am Acad Child Adolesc Psychiatry, 2019. **58**(5): p. 506-513.

13. Kothgassner, O.D., et al., *Does treatment method matter? A meta-analysis of the past 20 years of research on therapeutic interventions for self-harm and suicidal ideation in adolescents.* Borderline Personal Disord Emot Dysregul, 2020. **7**: p. 9.

For peer review only

For peer review only - http://bmjopen.bmj.com/site/about/guidelines.xhtml

14. Soto-Sanz, V., et al., *Internalizing and externalizing symptoms and suicidal behaviour in young people: a systematic review and meta-analysis of longitudinal studies.* Acta Psychiatr Scand, 2019. **140**(1): p. 5-19.

15. Melhem, N.M., et al., *Severity and Variability of Depression Symptoms Predicting Suicide Attempt in High-Risk Individuals.* JAMA Psychiatry, 2019. **76**(6): p. 603-613.

16. Borschmann, R., et al., *Self-harm in primary school-aged children: Prospective cohort study.* PLoS One, 2020. **15**(11): p. e0242802.

17. Miranda-Mendizabal, A., et al., *Gender differences in suicidal behavior in adolescents and young adults: systematic review and meta-analysis of longitudinal studies.* Int J Public Health, 2019. **64**(2): p. 265-283.

18. di Giacomo, E., et al., *Estimating the Risk of Attempted Suicide Among Sexual Minority Youths: A Systematic Review and Meta-analysis.* JAMA Pediatr, 2018. **172**(12): p. 1145-1152.

19. Liu, R.T., et al., *Negative life events and non-suicidal self-injury in an adolescent inpatient sample.* Arch Suicide Res, 2014. **18**(3): p. 251-8.

20. Gardvik, K.S., et al., *Suicidality, function and associated negative life events in an adolescent psychiatric population at 3-year follow-up.* BMC Psychiatry, 2021. **21**(1): p. 109.

21. Brent, D.A., et al., *Familial pathways to early-onset suicide attempt: a 5.6-year prospective study.* JAMA Psychiatry, 2015. **72**(2): p. 160-8.

22. Adrian, M., et al., *Predictors and moderators of recurring self-harm in adolescents participating in a comparative treatment trial of psychological interventions.* J Child Psychol Psychiatry, 2019. **60**(10): p. 1123-1132.

23. King, C.A., et al., *Family functioning and suicidal behavior in adolescent inpatients with mood disorders.* J Am Acad Child Adolesc Psychiatry, 1993. **32**(6): p. 1198-206.

For peer review only

For peer review only - http://bmjopen.bmj.com/site/about/guidelines.xhtml

24. Miller, A.B., C. Esposito-Smythers, and R.N. Leichtweis, *Role of social support in adolescent suicidal ideation and suicide attempts.* J Adolesc Health, 2015. **56**(3): p. 286-92.

25. Kushal, S.A., et al., *Parent-adolescent relationships and their associations with adolescent suicidal behaviours: Secondary analysis of data from 52 countries using the Global School-based Health Survey.* EClinicalMedicine, 2021. **31**: p. 100691.

26. Victor, S.E., et al., *Parent and peer relationships as longitudinal predictors of adolescent non-suicidal self-injury onset.* Child Adolesc Psychiatry Ment Health, 2019. **13**: p. 1.

27. Epstein, S., et al., *School absenteeism as a risk factor for self-harm and suicidal ideation in children and adolescents: a systematic review and meta-analysis.* Eur Child Adolesc Psychiatry, 2020. **29**(9): p. 1175-1194.

28. Orozco, R., et al., *Association between attempted suicide and academic performance indicators among middle and high school students in Mexico: results from a national survey.* Child Adolesc Psychiatry Ment Health, 2018. **12**: p. 9.

29. Goldstein, B.I., et al., *Inflammatory markers among adolescents and young adults with bipolar spectrum disorders.* J Clin Psychiatry, 2015. **76**(11): p. 1556-63.

30. Johnston, J.A.Y., et al., *Multimodal Neuroimaging of Frontolimbic Structure and Function Associated With Suicide Attempts in Adolescents and Young Adults With Bipolar Disorder.* Am J Psychiatry, 2017. **174**(7): p. 667-675.

31. Sarmiento-Hernández, E.I., et al., *Association between 5-HTTLPR polymorphism, suicide attempt and comorbidity in Mexican adolescents with major depressive disorder.* Actas Esp Psiquiatr, 2019. **47**(1): p. 1-6.

32. Zaborskis, A., et al., *The Association Between Cyberbullying, School Bullying, and Suicidality Among Adolescents.* Crisis, 2019. **40**(2): p. 100-114.

For peer review only - http://bmjopen.bmj.com/site/about/guidelines.xhtml

33. van Geel, M., P. Vedder, and J. Tanilon, *Relationship between peer victimization, cyberbullying, and suicide in children and adolescents: a meta-analysis.* JAMA Pediatr, 2014. **168**(5): p. 435-42.

34. McMahon, E.M., et al., *Factors associated with deliberate self-harm among Irish adolescents.* Psychol Med, 2010. **40**(11): p. 1811-9.

35. Sedgwick, R., et al., *Social media, internet use and suicide attempts in adolescents.* Curr Opin Psychiatry, 2019. **32**(6): p. 534-541.

36. Hawton, K., J. Fagg, and S. Simkin, *Deliberate self-poisoning and self-injury in children and adolescents under 16 years of age in Oxford, 1976-1993.* Br J Psychiatry, 1996. **169**(2): p. 202-8.

37. Consoli, A., et al., *Risk and Protective Factors for Suicidality at 6-Month Follow-up in Adolescent Inpatients Who Attempted Suicide: An Exploratory Model.* Can J Psychiatry, 2015. **60**(2 Suppl 1): p. S27-36.

38. Masi, G., et al., *Non suicidal self-injury in referred adolescents with mood disorders and its association with cyclothymic-hypersensitive temperament.* J Affect Disord, 2018. **227**: p. 477-482.

39. Russell, K., et al., *Sleep problem, suicide and self-harm in university students: A systematic review.* Sleep Med Rev, 2019. **44**: p. 58-69.

40. Fliege, H., et al., *Risk factors and correlates of deliberate self-harm behavior: a systematic review.* J Psychosom Res, 2009. **66**(6): p. 477-93.

41. Shamseer, L., et al., *Preferred reporting items for systematic review and meta-analysis protocols (PRISMA-P) 2015: elaboration and explanation.* BMJ, 2015. **350**: p. g7647.

42. Page, M.J., et al., *PRISMA 2020 explanation and elaboration: updated guidance and exemplars for reporting systematic reviews.* BMJ, 2021. **372**: p. n160.

For peer review only - http://bmjopen.bmj.com/site/about/guidelines.xhtml

43. Wilson, E. and D. Ougrin, *Commentary: Defining self-harm: how inconsistencies in language persist - a commentary/reflection on Ward and Curran (2021).* Child Adolesc Ment Health, 2021. **26**(4): p. 372-374.

44. International Society for the Study of Self-injury. *What is self-injury?* 2018; Available from: https://itriples.org/about-self-injury/what-is-self-injury.

45. Crosby, A., L. Ortega, and C. Melanson, *Self-directed violence surveillance: uniform definitions and recommended data elements (Version 1.0)*, C.f.D.C.a. Prevention and N.C.f.I.P.a. Control, Editors. 2011: Atlanta, Georgia.

46. Ougrin, D., et al., *Pandemic-related emergency psychiatric presentations for self-harm of children and adolescents in 10 countries (PREP-kids): a retrospective international cohort study.* European Child & Adolescent Psychiatry, 2022. **31**(7): p. 1-13.

47. Wright-Hughes, A., et al., *Reducing Self-harm in Adolescents. An individual participant data meta-analysis (RISA-IPD): systematic review protocol.* BMJ Open, 2021. **11**(5): p. e049255.

48. Minnis, H., R. Gajwani, and D. Ougrin, *Early intervention and prevention of severe mental illness: A child and adolescent psychiatry perspective.* Frontiers in Psychiatry, 2022. **13**.

49. BMJ Best Practice. *Study Design Search Filters*. Available from: https://bestpractice.bmj.com/info/toolkit/learn-ebm/study-design-search-filters/

50. Troy, D., et al., *What is the impact of structural and cultural factors and interventions within educational settings on promoting positive mental health and preventing poor mental health: a systematic review.* BMC Public Health, 2022. **22**(1): p. 524.

51. Baxter, S., et al., *The effects of integrated care: a systematic review of UK and international evidence.* BMC Health Serv Res, 2018. **18**(1): p. 350.

For peer review only

52. Leaviss, J., et al., *Behavioural modification interventions for medically unexplained symptoms in primary care: systematic reviews and economic evaluation.* Health Technol Assess, 2020. **24**(46): p. 1-490.

53. McHugh, M.L., *Interrater reliability: the kappa statistic.* Biochem Med (Zagreb), 2012. **22**(3): p. 276-82.

54. Wells, G.A., et al. *The Newcastle-Ottawa Scale (NOS) for assessing the quality of nonrandomised studies in meta-analyses*. Available from: https://www.ohri.ca/programs/clinical_epidemiology/oxford.asp.

55. Campbell, M., et al., *Synthesis without meta-analysis (SWiM) in systematic reviews: reporting guideline.* BMJ, 2020. **368**: p. l6890.

56. McKenzie J. E. and Brennan S. E., eds. *Synthesizing and presenting findings using other methods.* . Cochrane Handbook for Systematic Reviews of Interventions version 6.3 (updated February 2022), ed. Higgins J. P. T. , et al. 2022, Cochrane.

57. McHugh, M.L., *The odds ratio: calculation, usage and interpretation.* Biochem Med (Zagreb), 2009. **19**: p. 120-126.

58. Knol, M.J., A. Algra, and R.H. Groenwold, *How to deal with measures of association: a short guide for the clinician.* Cerebrovasc Dis, 2012. **33**(2): p. 98-103.

59. Page, M.J., J.P.T. Higgins, and J.A.C. Sterne, *Assessing risk of bias due to missing results in a synthesis.*, in *Cochrane Handbook for Systematic Reviews of Interventions version 6.3 (updated February 2022)*, J.P.T. Higgins, et al., Editors. 2022, Cochrane.

60. Guyatt, G.H., et al., *GRADE: an emerging consensus on rating quality of evidence and strength of recommendations.* BMJ, 2008. **336**(7650): p. 924-6.

61. Hetrick, S.E., et al., *Effective psychological and psychosocial approaches to reduce repetition of self-harm: a systematic review, meta-analysis and meta-regression.* BMJ Open, 2016. **6**(9): p. e011024.

For peer review only - http://bmjopen.bmj.com/site/about/guidelines.xhtml

62. Wang, J., et al., *Hypoglycemia and mortality in sepsis patients: A systematic review and meta-analysis.* Heart Lung, 2021. **50**(6): p. 933-940.

63. Mengist, B., et al., *Maternal near miss in Ethiopia: Protective role of antenatal care and disparity in socioeconomic inequities: A systematic review and meta-analysis.* International Journal of Africa Nursing Sciences, 2021. **15**: p. 100332.

For peer review only

For peer review only - http://bmjopen.bmj.com/site/about/guidelines.xhtml

PRISMA-P (Preferred Reporting Items for Systematic review and Meta-Analysis Protocols) 2015 checklist: recommended items to address in a systematic review protocol*

Section and topic	Item No	Checklist item	Page no.
**ADMINISTRATIVE INFORMATION**			
Title:			
Identification	1a	Identify the report as a protocol of a systematic review	Abstract, Main text P2.
Update	1b	If the protocol is for an update of a previous systematic review, identify as such	N/A
Registration	2	If registered, provide the name of the registry (such as PROSPERO) and registration number	Abstract, Main text P6.
Authors:			
Contact	3a	Provide name, institutional affiliation, e-mail address of all protocol authors; provide physical mailing address of corresponding author	Cover page P1.
Contributions	3b	Describe contributions of protocol authors and identify the guarantor of the review	P17.
Amendments	4	If the protocol represents an amendment of a previously completed or published protocol, identify as such and list changes; otherwise, state plan for documenting important protocol amendments	N/A
Support:			
Sources	5a	Indicate sources of financial or other support for the review	N/A
Sponsor	5b	Provide name for the review funder and/or sponsor	P17.
Role of sponsor or funder	5c	Describe roles of funder(s), sponsor(s), and/or institution(s), if any, in developing the protocol	N/A
**INTRODUCTION**			
Rationale	6	Describe the rationale for the review in the context of what is already known	P4-5
Objectives	7	Provide an explicit statement of the question(s) the review will address with reference to participants, interventions, comparators, and outcomes (PICO)	P5, Introduction last paragraph
**METHODS**			
Eligibility criteria	8	Specify the study characteristics (such as PICO, study design, setting, time frame) and report characteristics (such as years considered, language, publication status) to be used as criteria for eligibility for the review	P6-7
Information sources	9	Describe all intended information sources (such as electronic databases, contact with study authors, trial registers or other grey literature sources) with planned dates of coverage	P7-8
Search strategy	10	Present draft of search strategy to be used for at least one electronic database, including planned limits, such that it could be repeated	P26
Study records:			
Data management	11a	Describe the mechanism(s) that will be used to manage records and data throughout the review	P10 first paragraph, last line

For peer review only

Selection process	11b	State the process that will be used for selecting studies (such as two independent reviewers) through each phase of the review (that is, screening, eligibility and inclusion in meta-analysis)	P9-10
Data collection process	11c	Describe planned method of extracting data from reports (such as piloting forms, done independently, in duplicate), any processes for obtaining and confirming data from investigators	P10-11
Data items	12	List and define all variables for which data will be sought (such as PICO items, funding sources), any pre-planned data assumptions and simplifications	P10 bottom paragraph
Outcomes and prioritization	13	List and define all outcomes for which data will be sought, including prioritization of main and additional outcomes, with rationale	P11 first paragraph
Risk of bias in individual studies	14	Describe anticipated methods for assessing risk of bias of individual studies, including whether this will be done at the outcome or study level, or both; state how this information will be used in data synthesis	P11
Data synthesis	15a	Describe criteria under which study data will be quantitatively synthesised	P13 paragraph 2
	15b	If data are appropriate for quantitative synthesis, describe planned summary measures, methods of handling data and methods of combining data from studies, including any planned exploration of consistency (such as I^2, Kendall's τ)	P13 paragraphs 2-3
	15c	Describe any proposed additional analyses (such as sensitivity or subgroup analyses, meta-regression)	P14 paragraph 3
	15d	If quantitative synthesis is not appropriate, describe the type of summary planned	P12 paragraph 4
Meta-bias(es)	16	Specify any planned assessment of meta-bias(es) (such as publication bias across studies, selective reporting within studies)	P13-14
Confidence in cumulative evidence	17	Describe how the strength of the body of evidence will be assessed (such as GRADE)	P14 paragraph 2

*** It is strongly recommended that this checklist be read in conjunction with the PRISMA-P Explanation and Elaboration (cite when available) for important clarification on the items. Amendments to a review protocol should be tracked and dated. The copyright for PRISMA-P (including checklist) is held by the PRISMA-P Group and is distributed under a Creative Commons Attribution Licence 4.0.**

From: Shamseer L, Moher D, Clarke M, Ghersi D, Liberati A, Petticrew M, Shekelle P, Stewart L, PRISMA-P Group. Preferred reporting items for systematic review and meta-analysis protocols (PRISMA-P) 2015: elaboration and explanation. BMJ. 2015 Jan 2;349(jan02 1):g7647.

For peer review only

Example of search strategy (MEDLINE):

1. "Suicid*".mp. (mp=AB,OX,HW,FX,OT,SY,KF,PX,TI,NM,RX,UI)
2. "self injurious behavio?r*".mp.
3. "non-suicid*".mp.
4. NSSI.mp.
5. "Parasuicid*".mp.
6. "Self-injur*".mp.
7. "self-cut*".mp.
8. "self-harm*".mp.
9. "Self-Mutilat*".mp.
10. "Autoaggress*".mp.
11. "Automutilat*".mp.
12. "Self-destruct*".mp.
13. "Self-immolat*".mp.
14. "Self-poison*".mp.
15. "Self-inflict*".mp.
16. "Kill ????self".mp.
17. "Kill ????selves".mp.
18. or/1-17
19. "p?ediatr*".mp.
20. "adolescen*".mp.
21. "teen*".mp.
22. "child*".mp.
23. "youth*".mp.
24. "young people".mp.
25. "kid*".mp.
26. "girl*".mp.
27. "boy*".mp.
28. "pupil*".mp.
29. or/19-28
30. "risk*".mp.
31. exp risk factor/
32. exp association/
33. "causal*".mp.
34. "correlat*".mp.
35. "relat*".mp.

For peer review only

36. "associat*".mp.
37. "predict*".mp.
38. "Advers*".mp.
39. "antecedent*".mp.
40. "Histor*".mp.
41. "?etiolog*".mp.
42. "protect*".mp.
43. "prevent*".mp.
44. "improv*".mp.
45. prevalence.mp.
46. incidence.mp.
47. "rate*".mp.
48. hazard.mp.
49. or/30-48
50. "Longitudinal*".mp.
51. Prospective.mp.
52. epidemiolog*.mp.
53. retrospective.mp.
54. trajector*.mp.
55. "case control*".mp.
56. "follow-up".mp.
57. "follow up".mp.
58. "follow* up".mp.
59. "cohort*".mp.
60. "wave*".mp.
61. "time*".mp.
62. "multi-wave*".mp.
63. or/50-61
64. exp suicide/
65. exp self injurious behavior/
66. exp Suicide, Attempted/
67. exp Suicide, Completed/
68. exp self mutilation/
69. or/64-68
70. exp child/

For peer review only

71. exp adolescent/
72. 70 or 71
73. exp epidemiologic methods/
74. limit 73 to yr=1971-1988
75. exp Case-Control Studies/
76. exp epidemiological studies/
77. exp Cohort Studies/
78. exp Controlled Before-After Studies/
79. exp Follow-Up Studies/
80. exp Longitudinal Studies/
81. exp "national longitudinal study of adolescent health"/
82. exp Prospective Studies/
83. exp Retrospective Studies/
84. exp observational study/
85. exp Historically Controlled Study/
86. exp Interrupted Time Series Analysis/
87. or/74-86
88. 18 or 69
89. 29 or 72
90. 63 or 87
91. 49 and 88 and 89 and 90

For peer review only

BMJ Open

Risk and Protective Factors for Self-Harm and Suicide in Children and Adolescents: A Systematic Review and Meta-Analysis Protocol

Journal:	*BMJ Open*
Manuscript ID	bmjopen-2021-058297.R2
Article Type:	Protocol
Date Submitted by the Author:	31-Oct-2022

Complete List of Authors:	Farbstein, Dan; Rambam Health Care Campus, Child and Adolescent Psychiatry; Technion Israel Institute of Technology The Ruth and Bruce Rappaport Faculty of Medicine,<br>Lukito, Steve; King's College London, Department of Child & Adolescent Psychiatry, Institute of Psychiatry, Psychology and Neuroscience<br>Yorke, Isabel; King's College London, Department of Child & Adolescent Psychiatry, Institute of Psychiatry, Psychology and Neuroscience<br>Wilson, Emma; King's College London, Economic and Social Research Council (ESRC) Centre for Society and Mental Health; King's College London, Department of Health Service and Population Research, Institute of Psychiatry, Psychology and Neuroscience<br>Crudgington, Holly; King's College London, Economic and Social Research Council (ESRC) Centre for Society and Mental Health; King's College London, Department of Health Service and Population Research; Institute of Psychiatry, Psychology and Neuroscience<br>El-Aalem, Omar; South London and Maudsley NHS Foundation Trust<br>cliffe, charlotte; King's College London, Department of Biostatistics & Health Informatics, Institute of Psychiatry, Psychology and Neuroscience<br>Bergou, Nicol; King's College London, Department of Psychosis Studies, Institute of Psychiatry, Psychology and Neuroscience<br>Itani, Lynn; Maudsley Health, Al Amal Psychiatric Hospital, Emirates Health Services<br>Owusu, Andy; South London and Maudsley NHS Foundation Trust<br>Sedgwick, Rosemary; South London and Maudsley NHS Foundation Trust<br>Singh, Nidhita; South London and Maudsley NHS Foundation Trust<br>Tarasenko, Anna; BrainPatch Ltd<br>Tucker, Gavin; South London and Maudsley NHS Foundation Trust<br>Woodhouse, Emma; Compass Psychology Services Ltd; King's College London, Department of Forensic and Neurodevelopmental Sciences, Institute of Psychiatry, Psychology and Neuroscience,<br>Suzuki, Mimi; Queen Mary University of London & East London NHS Foundation Trust, Unit for Social and Community Psychiatry<br>Myerscough, Anna; Queen Mary University of London & East London NHS Foundation Trust, Unit for Social and Community Psychiatry<br>Lopez Chemas, Natalia; Queen Mary University of London & East London NHS Foundation Trust, Unit for Social and Community Psychiatry<br>Abdel-Halim, Nadia; Queen Mary University of London & East London NHS Foundation Trust, Unit for Social and Community Psychiatry<br>Del Giovane, Cinzia; University-Hospital of Modena and Reggio Emilia,

	Department of Medical and Surgical Sciences for Children and Adults; Institute of Primary Health Care (BIHAM), University of Bern Epstein, Sophie; King's College London, Department of Psychological Medicine, Institute of Psychiatry, Psychology and Neuroscience Ougrin, Dennis; King's College London, Department of Child & Adolescent Psychiatry, Institute of Psychiatry, Psychology and Neuroscience; Queen Mary University of London, Youth Resilience Research Unit, WHO Collaborating Centre for Mental Health Services Development
**Primary Subject Heading**:	Paediatrics
Secondary Subject Heading:	Mental health
Keywords:	Suicide & self-harm < PSYCHIATRY, Child & adolescent psychiatry < PSYCHIATRY, Community child health < PAEDIATRICS

SCHOLARONE™
Manuscripts

Risk and Protective Factors for Self-Harm and Suicide in Children and Adolescents: A Systematic Review and Meta-Analysis Protocol

Dan Farbstein[1,2†], Steve Lukito[3*†], Isabel Yorke[3], Emma Wilson[4,5], Holly Crudgington[4,5], Omar El-Aalem[6], Charlotte Cliffe[7,] Nicol Bergou[8], Lynn Itani[9], Andy Owusu[6], Rosemary Sedgwick[6], Nidhita Singh[6], Anna Tarasenko[10], Gavin Tucker[6], Emma Woodhouse[11, 12], Mimi Suzuki[13], Anna Louise Myerscough[13], Natalia Lopez Chemas[13], Nadia Abdel-Halim[13], Cinzia Del Giovane[14, 15], Sophie Epstein[16‡], Dennis Ougrin[17,3 ‡]

[1]Child and Adolescent Psychiatry Unit, Psychiatric Division, Rambam Healthcare Campus, Haifa, Israel; [2]Ruth and Bruce Rappaport Faculty of Medicine, Technion – Israeli Institute of Technology, Haifa, Israel; [3]Department of Child & Adolescent Psychiatry, Institute of Psychiatry, Psychology and Neuroscience, King's College London, United Kingdom; [4]Economic and Social Research Council (ESRC) Centre for Society and Mental Health, King's College London, United Kingdom; [5]Department of Health Service and Population Research, Institute of Psychiatry, Psychology and Neuroscience, King's College London, United Kingdom; [6]South London & Maudsley NHS Foundation Trust, United Kingdom; [7]Department of Biostatistics & Health Informatics, Institute of Psychiatry, Psychology and Neuroscience, King's College London, United Kingdom; [8]Department of Psychosis Studies, Institute of Psychiatry, Psychology and Neuroscience, King's College London, United Kingdom; [9]Maudsley Health, Al Amal Psychiatric Hospital, Emirates Health Services, Dubai, United Arab Emirates; [10]BrainPatch Ltd., London, United Kingdom; [11]Compass Psychology Services Ltd., Bromley, United Kingdom; [12]Department of Forensic and Neurodevelopmental Sciences, Institute of Psychiatry, Psychology and Neuroscience, King's College London, United Kingdom; [13] Unit for Social and Community Psychiatry, Queen Mary University of London & East London NHS Foundation Trust. [14]Department of Medical and Surgical Sciences for Children and Adults, University-Hospital of Modena and Reggio Emilia, Modena, Italy; [15]Institute of Primary Health Care (BIHAM), University of Bern, Switzerland, [16]Department of Psychological Medicine, Institute of Psychiatry, Psychology and Neuroscience, King's College London, United Kingdom; [17]Youth Resilience Research Unit, WHO Collaborating Centre for Mental Health Services Development, Queen Mary University of London, United Kingdom.

‡ Senior authors contributed equally to this work.

† Joint first author to this work

*Correspondence author: Steve Lukito, PhD.
King's College London
Institute of Psychiatry, Psychology and Neuroscience
Department of Child & Adolescent Psychiatry
De Crespigny Park
London SE5 8AF
Email: steve.lukito@kcl.ac.uk

For peer review only - http://bmjopen.bmj.com/site/about/guidelines.xhtml

Abstract:

Introduction: Self-harm and suicide are major public health concerns among children and adolescents. Many risk and protective factors for suicide and self-harm have been identified and reported in the literature. However, the capacity of these identified risk and protective factors to guide assessment and management is limited due to their great number. This protocol describes an ongoing systematic review and meta-analysis which aims to examine longitudinal studies of risk factors for self-harm and suicide in children and adolescents, to provide a comparison of the strengths of association of the various risk factors for self-harm and suicide and to shed light on those that require further investigation.

Methods and Analysis: We perform a systematic search of the literature using the databases EMBASE, PsycINFO, Medline, CINAHL and HMIC from inception up to 28 October 2020, and the search will be updated before the systematic review publication. Additionally, we will contact experts in the field, including principal investigators whose peer-reviewed publications are included in our systematic review as well as investigators from our extensive research network, and we will search the reference lists of relevant reviews to retrieve any articles that were not identified in our search. We will extract relevant data and present a narrative synthesis and combine the results in meta-analyses where there are sufficient data. We will assess the risk of bias for each study using the Newcastle-Ottawa Scale and present a summary of the quantity and the quality of the evidence for each risk or protective factor.

Ethics and dissemination: Ethical approval will not be sought as this is a systematic review of the literature. Results will be published in mental health journals and presented at conferences focused on suicide prevention.

PROSPERO registration number: CRD42021228212.

For peer review only - http://bmjopen.bmj.com/site/about/guidelines.xhtml

Strengths and limitations of this study:

- This systematic review will examine data about risk and protective factors for suicide and self-harm in children and adolescents only from longitudinal studies, thus avoiding issues of temporality that exist in cross-sectional studies.
- We will perform a thorough and systematic search including several databases from health and mental health fields, searching reference lists of previous systematic reviews and contacting key authors in the field.
- Using the broad definition of self-harm (as opposed to differentiating between suicide attempt and non-suicidal self-injury) may create challenges in synthesising the data as its definition in each study may be different, but on the other hand will prevent us from missing important evidence on the subject.
- Examination of all identified risk and protective factors for self-harm and suicide in children and adolescents simultaneously in one systematic review will allow comparison of the importance of each factor.
- This systematic review will only include studies published in the English language and therefore may overlook risk and protective factors that are unique to individual countries or parts of the world.

For peer review only

Introduction:

Suicide and self-harm, defined as any act of self-poisoning or self-injury, regardless of the motivation [1] among adolescents and young people are of major public health concern. Acts of self-harm are known to be highly prevalent in adolescents. International community-based studies estimate that approximately ten to thirty percent of adolescents self-harm [2, 3], and one to ten percent report to have made a suicide attempt at least once in their life [4-7]. These numbers are even higher in specific at-risk populations, such as those with psychiatric disorders, most notably depression and anxiety [6]. Most worryingly, self-harm and suicide attempts are some of the strongest predictive factors for completed, or death by, suicide [8, 9], which is now ranked as the second most common cause of death among 10-24 year olds, surpassed only by accidents [10].

Due to their great importance, many efforts have been undertaken to prevent self-harm and suicide attempts among adolescents. For example, a large international study examining school-based intervention programs for suicide prevention has showed that empowering youth and teaching coping skills results in decreased suicidal ideation and suicide attempts [11]. There have been recent advances in our understanding of precursors [12] and treatment [13] of self-harm in young people. However, the identification of adolescents at-risk for self-harm and suicide attempt remains difficult. Various studies and meta-analyses have been undertaken in an attempt to shed light on specific risk factors for self-harm and suicide in this population. Pre-existing externalising (e.g., conduct problems) and internalising symptoms (e.g., depression or anxiety) of psychopathology have been demonstrated to be a significant risk factors for suicidal behaviour [14, 15]. Other risk factors include: sociodemographic factors, such as age [16], gender, sexual orientation, transgender status and socioeconomic status [17, 18]; stressful life events, and most notably childhood abuse [19, 20]; familial factors, including familial psychopathology, family dysfunction, family conflict and perceived parental support

For peer review only - http://bmjopen.bmj.com/site/about/guidelines.xhtml

[21-26]; school-related factors such as academic performance and school attendance [27, 28]; biological factors such as genetic predisposition, changes in brain structure and function, and inflammatory status [29-31]; and social factors including peer-relatedness, bullying perpetration and victimisation, and social media use [16, 26, 32-36]. Some studies have examined the relationship between self-harm or suicidal behaviour and specific emotions, such as hopefulness and self-esteem and emotional lability [16, 34, 37, 38].

While these studies are of great importance in elucidating the risk factors for self-harm and suicidal behaviour, their clinical utility is limited. Such a large number of risk factors is likely to be overwhelming for the clinician meeting an adolescent in the emergency department, and in the primary care setting, and thus not a helpful guide for the psychiatric examination. Additionally, many systematic reviews and meta-analyses take into account both cross-sectional studies and longitudinal studies [see, e.g., 18, 27, 36, 39, 40]. Cross-sectional studies may be highly informative about the associations between various exposures and outcomes. However, risk or protective factors that are examined in cross-sectional studies cannot predict future events, due to the unclear temporal precedence between predictors and outcomes.

This protocol describes an ongoing systematic review and meta-analysis which will examine the evidence from longitudinal studies of all identified risk factors for self-harm and suicide in children and adolescents. This review will have two aims:

1. To provide summary strengths of association of the various risk factors for self-harm and suicide.
2. To identify risk factors for self-harm and suicide that are still debated in the literature, and thus to shed light on areas that are still in need of investigation.

Methods:

For peer review only - http://bmjopen.bmj.com/site/about/guidelines.xhtml

Our systematic review protocol follows the Preferred Reporting Items for Systematic Review and Meta-Analysis Protocols (PRISMA-P) guidelines [41] (online supplementary file 1—PRISMA-P checklist). The reporting of the systematic review and meta-analysis resulting from this protocol will follow the PRISMA2020 guidelines [42], and a completed PRISMA2020 checklist will be submitted with the publication. The protocol is registered on PROSPERO, the international register of systematic reviews (ID CRD42021228212). Any changes to the protocol will be recorded on PROSPERO.

Eligibility Criteria:

1. *Study Population:* Our target population is male or female children (i.e., young persons aged up to 10 years) and adolescents (i.e., young persons aged 11-17 years in early, mid and late adolescence). The population must be under the age of 18 years at the time of both exposure to the risk factor and outcome (self-harm or suicide). The decision to choose 18 years as the upper age limit was based on the fact that in most countries this is the age at which people transition to adulthood in many aspects. First, in many countries this is the age at which people complete school. Second, age 18 is when people legally become adults. Third, age 18 is the typical age at which the transition from child and adolescent to adult mental health services occurs. If a study includes both young persons under 18 and young adults who are over 18, then the study will only be included if (1) the average age of participants is under 18 or (2) more than 50% of the participants are under 18 or (3) there is a subgroup analysis for participants in the study meeting the criteria (1) or (2).
2. *Types of studies to be included:* We will include only quantitative longitudinal studies, either prospective or retrospective. Thus, cross-sectional studies will be excluded. Retrospective studies that measure the existence and extent of risk factors using recall will be excluded, to avoid bias which tends to result in the exaggeration of the

For peer review only - http://bmjopen.bmj.com/site/about/guidelines.xhtml

relationship between the risk factor and the outcome measured. As we consider many different risk/protective factors altogether, we do not set a threshold of eligibility for follow-up time in this study. Additionally, we will exclude intervention studies, qualitative studies, case reports or series, commentaries, editorials and conference abstracts. Only papers written in English language will be included.

3. *Exposure and outcome variables:* We will include any study that examines the relationship between any risk or protective factor and self-harm or suicide. Studies included must examine self-harm or suicide as an outcome. There are many definitions and subdivisions for self-harm in the literature. As mentioned above, for our study we decided to use the UK/European definition of self-harm, which is: any act of self-poisoning or self-injury carried out by a person, irrespective of their motivation [43]. This broad definition includes both (1) non-suicidal self-injury: self-inflicted destruction of body tissues without any suicidal intent [44], and (2) suicide attempts: a nonfatal self-directed potentially injurious behaviour with any intent to die as a result of the behaviour [45]. Any type of ascertainment of the outcome variables is acceptable, including but not limited to self-report in questionnaires or interviews, collateral report, school or police records and medical files. As suicidal ideation is not our outcome of interest, studies that report on suicidal ideation as the only outcome will be excluded.

Search Methods:

References will be extracted from the following databases according to the search strategy described below:

- Medline – Ovid Platform
- PsycINFO – Ovid platform

For peer review only - http://bmjopen.bmj.com/site/about/guidelines.xhtml

- EMBASE – Ovid platform
- HMIC – Ovid platform
- CINAHL – EBSCO platform.

Databases will be searched from inception, up to the present. The first wave of literature search was conducted to include studies published up to 28 October 2020, and the search will be updated before the systematic review publication.

For each of these databases, a search string was developed to include relevant keywords and synonyms (see "search strategy" in supplementary item 2). Examination of citation lists of previous relevant systematic reviews and meta-analyses will be conducted to retrieve any missed papers. Finally, experts in the field, which will include principal investigators whose peer-reviewed publications are included in our systematic review, as well as investigators from our extensive immediate research network [e.g., 35, 46, 47, 48], will be contacted to identify any missed papers.

Search strategy for electronic databases:

Scoping searches were initially performed to refine the search strategy and to optimise the balance between sensitivity and specificity. Search strategies were subsequently developed to include keywords and specific thesaurus terms for each database to include the following concepts:

1. Self-harm and suicide.
2. Populations under 18.
3. Association terms (e.g., relationship, risk factor, correlation, protective factors etc.)
4. Study designs.

To overcome linguistic variations, truncations and wildcards were employed when necessary. The list of keywords and thesaurus terms was based on previous systematic reviews about self-

For peer review only - http://bmjopen.bmj.com/site/about/guidelines.xhtml

harm and suicide [27, 35] and was discussed and finalised by a team of experts in the field of self-harm research in children and adolescents, tertiary- and secondary-level service clinicians, and experts in the fields of systematic reviews and meta-analyses. The concept of "study designs" was introduced in other systematic reviews in this field [14], and thus was employed in our search as well. For this concept we used the keywords and thesaurus terms recommended by the *British Medical Journal Best Practice* to identify cohort and case-control studies [49], but expanded the search to ensure sensitivity.

Reference Selection and Data Extraction:

Our research team includes 22 team members, comprising a mixture of researchers, clinicians and clinical academics, including several with expertise in self-harm and several with extensive experience of systematic reviews and meta-analyses including a senior statistician. Following removal of duplicate references, a two-staged screening of references is undertaken aided by a pre-specified screening instruction document. The document, which details the inclusion and exclusion criteria is devised as follows: After producing a first draft of the screening instructions, the entire team will screen 100 references with each member blinded to the decision of the other team members. The decisions will be discussed together in team meetings, and any ambiguity or lack of clarity in the screening instruction will be addressed and specific clarifications added to the instructions as necessary. This process will be repeated until a 90% concordance rate is reached among all team members.

Due to the anticipated large number of references and the constraint of time and resources to conduct this large-scale systematic review, once ≥ 90% concordance has been reached during the initial calibration process, remaining references will be divided equally between all team members. Following this process, 10% of all references will be screened by two independent reviewers and references will be equally divided into sets and be distributed among all team

For peer review only - http://bmjopen.bmj.com/site/about/guidelines.xhtml

members. Every set of references will be screened by one screener from the team during the title/abstract screening stage, with a random sample of 10% of the references reviewed by a second screener, following similar approaches in previous large-scale systematic reviews [e.g., 50, 51, 52]. Agreement rates for every pair of screeners will be recorded and reported as percent agreement [53]. If the agreement rates between pairs of screeners were lower than 90% within the set of 10% randomly selected articles, all remaining articles in this set will be double-screened. Articles progressing to full-text screening will be retrieved and screened by a single screener, with a random sample of 10% reviewed by a second screener, again similar to previous approaches [50-52]. If we identify more than one study which includes the same or overlapping samples, we will prioritise studies using the following hierarchy: largest sample, longest follow-up, adjusted for highest number of confounders. All discarded items will be recorded, including a reason for exclusion at the full-text screening stage. These screening stages will be conducted using the online systematic review tool Rayyan (https://www.rayyan.ai/).

Data will be extracted using a unified data extraction form that will be drafted *a priori* and collaboratively with the team. This will be piloted with the team and then for pragmatic reasons (due to the anticipated number of included studies) data on study characteristics will be extracted by single reviewers, with a random subsample of 10% of papers will be audited by a second reviewer. All statistical data relating to study results will be extracted by two independent reviewers. The following data will be extracted: type of study, participant eligibility and recruitment method, participant description including age, gender and ethnicity, diagnosis if applicable, country, study dates, sample size, definitions and measures of outcomes, blinding, duration of follow-up, number and type of predictors, definition and method for measurement of candidate predictors (e.g., categories may include demographics, psychopathology, family relationships, peer relationships, treatment history etc.), timing of

For peer review only - http://bmjopen.bmj.com/site/about/guidelines.xhtml

For peer review only

predictor measurement, missing data and its handling, authors, year of publication, type of statistical analysis, summary statistics and summary findings.

With regard to the summary statistics, we will collect effect measures expressing the association between risk factors and outcomes, as reported in individual studies, which are likely to include odds ratios (*OR*), hazard ratios (*HR*), risk ratios (*RR*), mean difference (*MD*) and standardised mean difference (*SMD*) with 95% confidence intervals (*CI*) and significance (*p*) values, or standard errors where appropriate, as well as which covariates have been adjusted for.

Any disagreement over reference eligibility, during the title/abstract and full-text screening stages, or data extraction will be discussed between the two screeners/reviewers. If a consensus cannot be reached, a third screener (DO) will adjudicate.

Risk of bias assessment

The Newcastle-Ottawa Scale (NOS) [54] will be used to assess risk of bias in observational/non-randomised studies. The risk of bias assessment will be shared out amongst multiple members of the research team with pairs of reviewers working independently applying the NOS. Separate versions of NOS are available for assessing the risks of bias in case-control and cohort studies, based on (1) the selection of study participants, e.g., their representativeness and ascertainment; (2) the comparability between participant groups, i.e., cases/exposed cohort or controls/non-exposed cohort; and (3) the risk of bias associated with the exposures in cohort studies, i.e., the ascertainment of exposures, comparability of exposure ascertainment in case and controls, and response rates; or outcomes in cohort studies, i.e., the assessments of outcomes, follow-up periods, and adequacy of follow-up of cohorts. Assessment of risk of bias will be conducted separately for case-control and cohort studies using the appropriate NOS versions.

For peer review only - http://bmjopen.bmj.com/site/about/guidelines.xhtml

Certain items of the NOS require tailoring to the specific systematic review or meta-analysis being conducted. A version of the NOS scale adapted for relevance to this research question has been developed and agreed a priori by the research team. In particular, we determined the acceptable level of loss to follow-up and acceptable methods of ascertainment of the exposure and outcome variables. Missing data within individual studies (e.g., due to attrition), will be reported and be taken into account as part of the quality assessment, and the level of bias it introduced into the findings will be considered for each study.

Finally, the discrepancies between two reviewers regarding a study's risk of bias ratings will be resolved through discussions, if necessary, in the presence of a third reviewer (DO) who will adjudicate if consensus cannot be reached.

Data presentation and statistical analysis:

Where possible a meta-analysis will be conducted to synthesise evidence. Alternatively, we will perform a narrative synthesis without meta-analysis using the quantitative data of the included studies [55]. Depending on the available data, synthesis of quantitative data may involve summarising estimates of effect (i.e., strength of association in our context) in narratives, or vote counting based on the direction of the effects [56]. We will calculate missing outcome data, if possible, from the available data within the article to enable consistent reporting or potentially to include them in the meta-analyses. If this is not possible, the findings will be reported within the narrative synthesis in qualitative manner. The findings of association between risk factors and self-harm outcomes will be grouped according to categories of outcomes (i.e., self-harm or completed suicide). Then, for each outcome group, the association between the risk factors and the outcome will be grouped by similar risk-factor concepts, in line with clinically meaningful categorisation from past literature, for instance 'existing psychopathology', including internalising, externalising, and general

For peer review only - http://bmjopen.bmj.com/site/about/guidelines.xhtml

psychopathology; ‘family relationships’, including family dynamics, parenting styles and familial resources; ‘demographic variables’, including socioeconomic status, ethnicity, sexual orientation and gender identity; ‘peer relationships’, such as bullying and victimisation, ostracisation, and interactions via social media; and previous self-harm events. The relative strength of associations between all identified factors with the outcome will be discussed. Extracted data about participant sample characteristics, study methodologies, and a summary of findings from each study will be presented in a table of characteristics of included studies.

Random-effect meta-analysis will be conducted, given the anticipated variation of sample characteristics and outcome measures across studies. There is likely to be variation in the statistical effect measures used across the literature, however, most studies are expected to compare those with and without the exposure of interest when estimating their association with binary self-harm outcomes (i.e., with or without self-harm), and would use *OR*s, *HR*s or *RR*s to index such association. *OR*s are expected to be the effect measure mostly used by any eligible case-control studies. Furthermore, the *OR*s can be easily interpreted clinically [57, 58]. For these reasons, synthesis will be conducted using *OR* summary statistics to examine the pooled association between each predictor and self-harm. Where possible, other effect measures will be transformed to *OR*s for synthesis.

The meta-analyses will be conducted on findings from studies investigating similar exposure and outcome variables. If a study reports multiple effects of interest, then we will include all the available effects. If there is more than one reported effect in relation to the same exposure and outcome, the effect adjusted for the largest number of confounders will be included. We will illustrate the data using a forest plot, with 95% *CI* and *p*-value reported. It is expected that there will be significant heterogeneity between studies. Thus, the I^2 statistics will be calculated to index statistical heterogeneity in the meta-analysis. Finally, publication bias will

For peer review only - http://bmjopen.bmj.com/site/about/guidelines.xhtml

be assessed by funnel plots and Egger's test if there are at least ten studies within the meta-analysis (Cochrane Handbook 13.3.5.4; [59]).

For each exposure and outcome, an assessment of strength of evidence will be performed by applying the Grading of Recommendations Assessment, Development and Evaluation (GRADE; [60]) approach adapted for non-randomised studies. We will use the GRADE tool to assess the quality of evidence, i.e., the certainty of the estimate of association between individual risk factors and each self-harm outcome. The GRADE tool will only be applied following a meta-analysis, i.e., when the pooled evidence of association between specific risk factors and a self-harm outcome could be obtained. We will use the 95% *CI* to assess precision for the GRADE tool. If the 95% *CI* does not cross the line of non-significance, the risk estimate will be considered to be sufficiently precise.

Subgroup analyses according to age (children, i.e., young persons aged up to 10 years *vs.* adolescents, i.e., young persons aged 11-17 years), gender (female *vs.* male) and country (high- *vs.* low- and middle-income country) will be conducted if possible. The quantitative synthesis of findings will be based on meta-analyses of all samples collected in the study. However, to assess the influence of risk of bias of the individual studies in the quantitative synthesis, we also plan to conduct sensitivity meta-analyses including only studies deemed to have low risk of bias (see e.g. [61] for a similar approach). Studies with low risk of bias are defined by a total NOS score ≥ 7 following past reports [e.g., 62, 63].

Patient and Public Involvement: Not applicable.

Discussion:

This systematic review and meta-analysis will attempt to outline the risk and protective factors for self-harm and suicide among children and adolescents. A major strength of our work will be its focus on longitudinal studies. The exclusion of cross-sectional studies will highlight the

For peer review only - http://bmjopen.bmj.com/site/about/guidelines.xhtml

risk factors and protective factors that predict self-harm and suicide, as opposed to those that co-occur with them.

Conducting this systematic review and meta-analysis presents various challenges. Although we have used broad search terms to increase the chance of identifying all relevant data, there remains a chance that data could be missed due to the wide range of different terminology used in different settings. Additionally, it is possible that suicide or self-harm may not be reported in the title or abstract and are omitted from the keywords if it is not a major outcome in the study. To reduce the risk of missing significant data, in addition to database searches, we will examine the reference lists of previous systematic reviews and meta-analyses on the subject and consult experts in the field (i.e., principal investigators of peer-reviewed papers included in this review as well as investigators from our research network) to identify additional papers. An additional challenge will be the synthesis of the data. Besides the variation in the level of bias in the studies that needs to be accounted for, methodology may differ significantly for the measurement of different risk and protective factors. For example, psychiatric symptoms may be measured using various methods: clinical evaluation, questionnaires and structured or semi-structured interviews, each method with its unique features in terms of reliability and validity. These variations will need to be taken into account when synthesising data of different studies.

The primary goal of our work will be to describe the strength of association between various risk and protective factors and self-harm and suicide outcomes in children and adolescents. We hope this work will inform the clinician assessing a child or adolescent in the outpatient clinic or emergency department regarding important areas that need to be covered to optimally assess future self-harm and suicide risk. The need for future research in different areas, whether due to insufficient studies, poorly conducted studies, or the lack of use of similar terminology or methodology that would allow adequate data synthesis and conclusions, will also be highlighted.

For peer review only - http://bmjopen.bmj.com/site/about/guidelines.xhtml

Ethics and dissemination:

Ethical approval for this study is not needed, since it is based on the review of published literature only. The full protocol and the findings of this study will be published in peer-reviewed journals whose major audience are practitioners in child and adolescent mental health.

For peer review only

Authors contributions:

Dan Farbstein, Steve Lukito: Drafting of manuscript, designing of study, search strategy, screening process and analysis.

Isabel Yorke: Designing of study, search strategy, screening process and analysis, critical revision and review of the protocol.

Emma Wilson, Holly Crudgington, Omar El-Aalem, Nicol Bergou, Charlotte Cliffe, Lynn Itani, Andy Owusu, Rosemary Sedgwick, Nidhita Singh, Anna Tarasenko, Gavin Tucker, Emma Woodhouse: Designing of study, search strategy, screening process and analysis, revision and review of protocol.

Mimi Suzuki, Anna Louise Myerscough, Natalia Lopez Chemas, Nadia Abdel-Halim: Screening process and analysis, revision and review of protocol.

Cinzia Del Giovane, Sophie Epstein, Dennis Ougrin: Designing of study, search strategy, screening process and analysis, critical revision and review of protocol.

Funding statement:

This work was supported by the joint UK Medical Research Foundation [MRF-058-0005-RG-OUGRI] and Medical Research Council (MRC) [MR/R004927/1] grant to DO, and the Daniel Turnberg Travel Fellowship [DTTFR12\1055] to DF. EW and HC were supported by the Economic and Social Research Council (ESRC) Centre for Society and Mental Health at King's College London [ES/S012567/1]. SE was funded by an MRC Clinical Research Training Fellowship [MR/T001437/1] and previously received salary support from an MQ Data Science Award and from the Psychiatry Research Trust. This work was also supported by funding from the UK Department of Health via the National Institute for Health Research

For peer review only - http://bmjopen.bmj.com/site/about/guidelines.xhtml

(NIHR) Biomedical Research Centre (BRC) for Mental Health at South London (grant number N/A) and the Maudsley National Health Service (NHS) Foundation Trust and the IoPPN, King's College London (grant number N/A). The views expressed are those of the authors and not necessarily those of the NHS, the NIHR, MRC, MRF, ESRC, or the Department of Health.

Competing interests:

The authors have no competing interests to disclose.

Word count: 3634

References:

1. National Institute for Health and Care Excellence (NICE), *Self-harm [NICE Quality Standard No. 34]*. 2019.
2. Brunner, R., et al., *Life-time prevalence and psychosocial correlates of adolescent direct self-injurious behavior: a comparative study of findings in 11 European countries.* J Child Psychol Psychiatry, 2014. **55**(4): p. 337-48.
3. Hawton, K., K.E. Saunders, and R.C. O'Connor, *Self-harm and suicide in adolescents.* Lancet, 2012. **379**(9834): p. 2373-82.
4. Madge, N., et al., *Deliberate self-harm within an international community sample of young people: comparative findings from the Child & Adolescent Self-harm in Europe (CASE) Study.* J Child Psychol Psychiatry, 2008. **49**(6): p. 667-77.

For peer review only - http://bmjopen.bmj.com/site/about/guidelines.xhtml

5. Nock, M.K., et al., *Prevalence, correlates, and treatment of lifetime suicidal behavior among adolescents: results from the National Comorbidity Survey Replication Adolescent Supplement.* JAMA Psychiatry, 2013. **70**(3): p. 300-10.

6. Moran, P., et al., *The natural history of self-harm from adolescence to young adulthood: a population-based cohort study.* Lancet, 2012. **379**(9812): p. 236-43.

7. Kokkevi, A., et al., *Adolescents' self-reported suicide attempts, self-harm thoughts and their correlates across 17 European countries.* J Child Psychol Psychiatry, 2012. **53**(4): p. 381-9.

8. Hawton, K. and L. Harriss, *Deliberate self-harm in young people: characteristics and subsequent mortality in a 20-year cohort of patients presenting to hospital.* J Clin Psychiatry, 2007. **68**(10): p. 1574-83.

9. Joiner, T.E., Jr., et al., *Four studies on how past and current suicidality relate even when "everything but the kitchen sink" is covaried.* J Abnorm Psychol, 2005. **114**(2): p. 291-303.

10. Patton, G.C., et al., *Global patterns of mortality in young people: a systematic analysis of population health data.* Lancet, 2009. **374**(9693): p. 881-92.

11. Wasserman, D., et al., *School-based suicide prevention programmes: the SEYLE cluster-randomised, controlled trial.* Lancet, 2015. **385**(9977): p. 1536-44.

12. Baldwin, J.R., et al., *Adolescent Victimization and Self-Injurious Thoughts and Behaviors: A Genetically Sensitive Cohort Study.* J Am Acad Child Adolesc Psychiatry, 2019. **58**(5): p. 506-513.

13. Kothgassner, O.D., et al., *Does treatment method matter? A meta-analysis of the past 20 years of research on therapeutic interventions for self-harm and suicidal ideation in adolescents.* Borderline Personal Disord Emot Dysregul, 2020. **7**: p. 9.

For peer review only - http://bmjopen.bmj.com/site/about/guidelines.xhtml

14. Soto-Sanz, V., et al., *Internalizing and externalizing symptoms and suicidal behaviour in young people: a systematic review and meta-analysis of longitudinal studies.* Acta Psychiatr Scand, 2019. **140**(1): p. 5-19.

15. Melhem, N.M., et al., *Severity and Variability of Depression Symptoms Predicting Suicide Attempt in High-Risk Individuals.* JAMA Psychiatry, 2019. **76**(6): p. 603-613.

16. Borschmann, R., et al., *Self-harm in primary school-aged children: Prospective cohort study.* PLoS One, 2020. **15**(11): p. e0242802.

17. Miranda-Mendizabal, A., et al., *Gender differences in suicidal behavior in adolescents and young adults: systematic review and meta-analysis of longitudinal studies.* Int J Public Health, 2019. **64**(2): p. 265-283.

18. di Giacomo, E., et al., *Estimating the Risk of Attempted Suicide Among Sexual Minority Youths: A Systematic Review and Meta-analysis.* JAMA Pediatr, 2018. **172**(12): p. 1145-1152.

19. Liu, R.T., et al., *Negative life events and non-suicidal self-injury in an adolescent inpatient sample.* Arch Suicide Res, 2014. **18**(3): p. 251-8.

20. Gardvik, K.S., et al., *Suicidality, function and associated negative life events in an adolescent psychiatric population at 3-year follow-up.* BMC Psychiatry, 2021. **21**(1): p. 109.

21. Brent, D.A., et al., *Familial pathways to early-onset suicide attempt: a 5.6-year prospective study.* JAMA Psychiatry, 2015. **72**(2): p. 160-8.

22. Adrian, M., et al., *Predictors and moderators of recurring self-harm in adolescents participating in a comparative treatment trial of psychological interventions.* J Child Psychol Psychiatry, 2019. **60**(10): p. 1123-1132.

23. King, C.A., et al., *Family functioning and suicidal behavior in adolescent inpatients with mood disorders.* J Am Acad Child Adolesc Psychiatry, 1993. **32**(6): p. 1198-206.

For peer review only

For peer review only - http://bmjopen.bmj.com/site/about/guidelines.xhtml

24. Miller, A.B., C. Esposito-Smythers, and R.N. Leichtweis, *Role of social support in adolescent suicidal ideation and suicide attempts.* J Adolesc Health, 2015. **56**(3): p. 286-92.

25. Kushal, S.A., et al., *Parent-adolescent relationships and their associations with adolescent suicidal behaviours: Secondary analysis of data from 52 countries using the Global School-based Health Survey.* EClinicalMedicine, 2021. **31**: p. 100691.

26. Victor, S.E., et al., *Parent and peer relationships as longitudinal predictors of adolescent non-suicidal self-injury onset.* Child Adolesc Psychiatry Ment Health, 2019. **13**: p. 1.

27. Epstein, S., et al., *School absenteeism as a risk factor for self-harm and suicidal ideation in children and adolescents: a systematic review and meta-analysis.* Eur Child Adolesc Psychiatry, 2020. **29**(9): p. 1175-1194.

28. Orozco, R., et al., *Association between attempted suicide and academic performance indicators among middle and high school students in Mexico: results from a national survey.* Child Adolesc Psychiatry Ment Health, 2018. **12**: p. 9.

29. Goldstein, B.I., et al., *Inflammatory markers among adolescents and young adults with bipolar spectrum disorders.* J Clin Psychiatry, 2015. **76**(11): p. 1556-63.

30. Johnston, J.A.Y., et al., *Multimodal Neuroimaging of Frontolimbic Structure and Function Associated With Suicide Attempts in Adolescents and Young Adults With Bipolar Disorder.* Am J Psychiatry, 2017. **174**(7): p. 667-675.

31. Sarmiento-Hernández, E.I., et al., *Association between 5-HTTLPR polymorphism, suicide attempt and comorbidity in Mexican adolescents with major depressive disorder.* Actas Esp Psiquiatr, 2019. **47**(1): p. 1-6.

32. Zaborskis, A., et al., *The Association Between Cyberbullying, School Bullying, and Suicidality Among Adolescents.* Crisis, 2019. **40**(2): p. 100-114.

For peer review only - http://bmjopen.bmj.com/site/about/guidelines.xhtml

33. van Geel, M., P. Vedder, and J. Tanilon, *Relationship between peer victimization, cyberbullying, and suicide in children and adolescents: a meta-analysis.* JAMA Pediatr, 2014. **168**(5): p. 435-42.

34. McMahon, E.M., et al., *Factors associated with deliberate self-harm among Irish adolescents.* Psychol Med, 2010. **40**(11): p. 1811-9.

35. Sedgwick, R., et al., *Social media, internet use and suicide attempts in adolescents.* Curr Opin Psychiatry, 2019. **32**(6): p. 534-541.

36. Hawton, K., J. Fagg, and S. Simkin, *Deliberate self-poisoning and self-injury in children and adolescents under 16 years of age in Oxford, 1976-1993.* Br J Psychiatry, 1996. **169**(2): p. 202-8.

37. Consoli, A., et al., *Risk and Protective Factors for Suicidality at 6-Month Follow-up in Adolescent Inpatients Who Attempted Suicide: An Exploratory Model.* Can J Psychiatry, 2015. **60**(2 Suppl 1): p. S27-36.

38. Masi, G., et al., *Non suicidal self-injury in referred adolescents with mood disorders and its association with cyclothymic-hypersensitive temperament.* J Affect Disord, 2018. **227**: p. 477-482.

39. Russell, K., et al., *Sleep problem, suicide and self-harm in university students: A systematic review.* Sleep Med Rev, 2019. **44**: p. 58-69.

40. Fliege, H., et al., *Risk factors and correlates of deliberate self-harm behavior: a systematic review.* J Psychosom Res, 2009. **66**(6): p. 477-93.

41. Shamseer, L., et al., *Preferred reporting items for systematic review and meta-analysis protocols (PRISMA-P) 2015: elaboration and explanation.* BMJ, 2015. **350**: p. g7647.

42. Page, M.J., et al., *PRISMA 2020 explanation and elaboration: updated guidance and exemplars for reporting systematic reviews.* BMJ, 2021. **372**: p. n160.

For peer review only - http://bmjopen.bmj.com/site/about/guidelines.xhtml

43. Wilson, E. and D. Ougrin, *Commentary: Defining self-harm: how inconsistencies in language persist - a commentary/reflection on Ward and Curran (2021).* Child Adolesc Ment Health, 2021. **26**(4): p. 372-374.

44. International Society for the Study of Self-injury. *What is self-injury?* 2018; Available from: https://itriples.org/about-self-injury/what-is-self-injury.

45. Crosby, A., L. Ortega, and C. Melanson, *Self-directed violence surveillance: uniform definitions and recommended data elements (Version 1.0)*, C.f.D.C.a. Prevention and N.C.f.I.P.a. Control, Editors. 2011: Atlanta, Georgia.

46. Ougrin, D., et al., *Pandemic-related emergency psychiatric presentations for self-harm of children and adolescents in 10 countries (PREP-kids): a retrospective international cohort study.* European Child & Adolescent Psychiatry, 2022. **31**(7): p. 1-13.

47. Wright-Hughes, A., et al., *Reducing Self-harm in Adolescents. An individual participant data meta-analysis (RISA-IPD): systematic review protocol.* BMJ Open, 2021. **11**(5): p. e049255.

48. Minnis, H., R. Gajwani, and D. Ougrin, *Early intervention and prevention of severe mental illness: A child and adolescent psychiatry perspective.* Frontiers in Psychiatry, 2022. **13**.

49. BMJ Best Practice. *Study Design Search Filters*. Available from: https://bestpractice.bmj.com/info/toolkit/learn-ebm/study-design-search-filters/

50. Troy, D., et al., *What is the impact of structural and cultural factors and interventions within educational settings on promoting positive mental health and preventing poor mental health: a systematic review.* BMC Public Health, 2022. **22**(1): p. 524.

51. Baxter, S., et al., *The effects of integrated care: a systematic review of UK and international evidence.* BMC Health Serv Res, 2018. **18**(1): p. 350.

For peer review only - http://bmjopen.bmj.com/site/about/guidelines.xhtml

52. Leaviss, J., et al., *Behavioural modification interventions for medically unexplained symptoms in primary care: systematic reviews and economic evaluation.* Health Technol Assess, 2020. **24**(46): p. 1-490.

53. McHugh, M.L., *Interrater reliability: the kappa statistic.* Biochem Med (Zagreb), 2012. **22**(3): p. 276-82.

54. Wells, G.A., et al. *The Newcastle-Ottawa Scale (NOS) for assessing the quality of nonrandomised studies in meta-analyses*. Available from: https://www.ohri.ca/programs/clinical_epidemiology/oxford.asp.

55. Campbell, M., et al., *Synthesis without meta-analysis (SWiM) in systematic reviews: reporting guideline.* BMJ, 2020. **368**: p. l6890.

56. McKenzie J. E. and Brennan S. E., eds. *Synthesizing and presenting findings using other methods.* . Cochrane Handbook for Systematic Reviews of Interventions version 6.3 (updated February 2022), ed. Higgins J. P. T. , et al. 2022, Cochrane.

57. McHugh, M.L., *The odds ratio: calculation, usage and interpretation.* Biochem Med (Zagreb), 2009. **19**: p. 120-126.

58. Knol, M.J., A. Algra, and R.H. Groenwold, *How to deal with measures of association: a short guide for the clinician.* Cerebrovasc Dis, 2012. **33**(2): p. 98-103.

59. Page, M.J., J.P.T. Higgins, and J.A.C. Sterne, *Assessing risk of bias due to missing results in a synthesis.*, in *Cochrane Handbook for Systematic Reviews of Interventions version 6.3 (updated February 2022)*, J.P.T. Higgins, et al., Editors. 2022, Cochrane.

60. Guyatt, G.H., et al., *GRADE: an emerging consensus on rating quality of evidence and strength of recommendations.* BMJ, 2008. **336**(7650): p. 924-6.

61. Hetrick, S.E., et al., *Effective psychological and psychosocial approaches to reduce repetition of self-harm: a systematic review, meta-analysis and meta-regression.* BMJ Open, 2016. **6**(9): p. e011024.

For peer review only - http://bmjopen.bmj.com/site/about/guidelines.xhtml

62. Wang, J., et al., *Hypoglycemia and mortality in sepsis patients: A systematic review and meta-analysis.* Heart Lung, 2021. **50**(6): p. 933-940.

63. Mengist, B., et al., *Maternal near miss in Ethiopia: Protective role of antenatal care and disparity in socioeconomic inequities: A systematic review and meta-analysis.* International Journal of Africa Nursing Sciences, 2021. **15**: p. 100332.

For peer review only

For peer review only - http://bmjopen.bmj.com/site/about/guidelines.xhtml

PRISMA-P (Preferred Reporting Items for Systematic review and Meta-Analysis Protocols) 2015 checklist: recommended items to address in a systematic review protocol*

Section and topic	Item No	Checklist item	Page no.
**ADMINISTRATIVE INFORMATION**			
Title:			
Identification	1a	Identify the report as a protocol of a systematic review	Abstract, Main text P2.
Update	1b	If the protocol is for an update of a previous systematic review, identify as such	N/A
Registration	2	If registered, provide the name of the registry (such as PROSPERO) and registration number	Abstract, Main text P6.
Authors:			
Contact	3a	Provide name, institutional affiliation, e-mail address of all protocol authors; provide physical mailing address of corresponding author	Cover page P1.
Contributions	3b	Describe contributions of protocol authors and identify the guarantor of the review	P17.
Amendments	4	If the protocol represents an amendment of a previously completed or published protocol, identify as such and list changes; otherwise, state plan for documenting important protocol amendments	N/A
Support:			
Sources	5a	Indicate sources of financial or other support for the review	N/A
Sponsor	5b	Provide name for the review funder and/or sponsor	P17.
Role of sponsor or funder	5c	Describe roles of funder(s), sponsor(s), and/or institution(s), if any, in developing the protocol	N/A
**INTRODUCTION**			
Rationale	6	Describe the rationale for the review in the context of what is already known	P4-5
Objectives	7	Provide an explicit statement of the question(s) the review will address with reference to participants, interventions, comparators, and outcomes (PICO)	P5, Introduction last paragraph
**METHODS**			
Eligibility criteria	8	Specify the study characteristics (such as PICO, study design, setting, time frame) and report characteristics (such as years considered, language, publication status) to be used as criteria for eligibility for the review	P6-7
Information sources	9	Describe all intended information sources (such as electronic databases, contact with study authors, trial registers or other grey literature sources) with planned dates of coverage	P7-8
Search strategy	10	Present draft of search strategy to be used for at least one electronic database, including planned limits, such that it could be repeated	P26
Study records:			
Data management	11a	Describe the mechanism(s) that will be used to manage records and data throughout the review	P10 first paragraph, last line

For peer review only - http://bmjopen.bmj.com/site/about/guidelines.xhtml

Selection process	11b	State the process that will be used for selecting studies (such as two independent reviewers) through each phase of the review (that is, screening, eligibility and inclusion in meta-analysis)	P9-10
Data collection process	11c	Describe planned method of extracting data from reports (such as piloting forms, done independently, in duplicate), any processes for obtaining and confirming data from investigators	P10-11
Data items	12	List and define all variables for which data will be sought (such as PICO items, funding sources), any pre-planned data assumptions and simplifications	P10 bottom paragraph
Outcomes and prioritization	13	List and define all outcomes for which data will be sought, including prioritization of main and additional outcomes, with rationale	P11 first paragraph
Risk of bias in individual studies	14	Describe anticipated methods for assessing risk of bias of individual studies, including whether this will be done at the outcome or study level, or both; state how this information will be used in data synthesis	P11
Data synthesis	15a	Describe criteria under which study data will be quantitatively synthesised	P13 paragraph 2
	15b	If data are appropriate for quantitative synthesis, describe planned summary measures, methods of handling data and methods of combining data from studies, including any planned exploration of consistency (such as I^2, Kendall's τ)	P13 paragraphs 2-3
	15c	Describe any proposed additional analyses (such as sensitivity or subgroup analyses, meta-regression)	P14 paragraph 3
	15d	If quantitative synthesis is not appropriate, describe the type of summary planned	P12 paragraph 4
Meta-bias(es)	16	Specify any planned assessment of meta-bias(es) (such as publication bias across studies, selective reporting within studies)	P13-14
Confidence in cumulative evidence	17	Describe how the strength of the body of evidence will be assessed (such as GRADE)	P14 paragraph 2

*** It is strongly recommended that this checklist be read in conjunction with the PRISMA-P Explanation and Elaboration (cite when available) for important clarification on the items. Amendments to a review protocol should be tracked and dated. The copyright for PRISMA-P (including checklist) is held by the PRISMA-P Group and is distributed under a Creative Commons Attribution Licence 4.0.**

From: Shamseer L, Moher D, Clarke M, Ghersi D, Liberati A, Petticrew M, Shekelle P, Stewart L, PRISMA-P Group. Preferred reporting items for systematic review and meta-analysis protocols (PRISMA-P) 2015: elaboration and explanation. BMJ. 2015 Jan 2;349(jan02 1):g7647.

For peer review only

For peer review only - http://bmjopen.bmj.com/site/about/guidelines.xhtml

Example of search strategy (MEDLINE):

1. "Suicid*".mp. (mp=AB,OX,HW,FX,OT,SY,KF,PX,TI,NM,RX,UI)
2. "self injurious behavio?r*".mp.
3. "non-suicid*".mp.
4. NSSI.mp.
5. "Parasuicid*".mp.
6. "Self-injur*".mp.
7. "self-cut*".mp.
8. "self-harm*".mp.
9. "Self-Mutilat*".mp.
10. "Autoaggress*".mp.
11. "Automutilat*".mp.
12. "Self-destruct*".mp.
13. "Self-immolat*".mp.
14. "Self-poison*".mp.
15. "Self-inflict*".mp.
16. "Kill ????self".mp.
17. "Kill ????selves".mp.
18. or/1-17
19. "p?ediatr*".mp.
20. "adolescen*".mp.
21. "teen*".mp.
22. "child*".mp.
23. "youth*".mp.
24. "young people".mp.
25. "kid*".mp.
26. "girl*".mp.
27. "boy*".mp.
28. "pupil*".mp.
29. or/19-28
30. "risk*".mp.
31. exp risk factor/
32. exp association/
33. "causal*".mp.
34. "correlat*".mp.
35. "relat*".mp.

For peer review only

36. "associat*".mp.
37. "predict*".mp.
38. "Advers*".mp.
39. "antecedent*".mp.
40. "Histor*".mp.
41. "?etiolog*".mp.
42. "protect*".mp.
43. "prevent*".mp.
44. "improv*".mp.
45. prevalence.mp.
46. incidence.mp.
47. "rate*".mp.
48. hazard.mp.
49. or/30-48
50. "Longitudinal*".mp.
51. Prospective.mp.
52. epidemiolog*.mp.
53. retrospective.mp.
54. trajector*.mp.
55. "case control*".mp.
56. "follow-up".mp.
57. "follow up".mp.
58. "follow* up".mp.
59. "cohort*".mp.
60. "wave*".mp.
61. "time*".mp.
62. "multi-wave*".mp.
63. or/50-61
64. exp suicide/
65. exp self injurious behavior/
66. exp Suicide, Attempted/
67. exp Suicide, Completed/
68. exp self mutilation/
69. or/64-68
70. exp child/

For peer review only

71. exp adolescent/
72. 70 or 71
73. exp epidemiologic methods/
74. limit 73 to yr=1971-1988
75. exp Case-Control Studies/
76. exp epidemiological studies/
77. exp Cohort Studies/
78. exp Controlled Before-After Studies/
79. exp Follow-Up Studies/
80. exp Longitudinal Studies/
81. exp "national longitudinal study of adolescent health"/
82. exp Prospective Studies/
83. exp Retrospective Studies/
84. exp observational study/
85. exp Historically Controlled Study/
86. exp Interrupted Time Series Analysis/
87. or/74-86
88. 18 or 69
89. 29 or 72
90. 63 or 87
91. 49 and 88 and 89 and 90

For peer review only
